# Proper RPA acetylation promotes accurate DNA replication and repair

**DOI:** 10.1093/nar/gkad291

**Published:** 2023-05-04

**Authors:** Xiaoli Gan, Yueyue Zhang, Donghao Jiang, Jingyao Shi, Han Zhao, Chengyu Xie, Yanyan Wang, Jingyan Xu, Xinghua Zhang, Gang Cai, Hailong Wang, Jun Huang, Xuefeng Chen

**Affiliations:** Hubei Key Laboratory of Cell Homeostasis, College of Life Sciences, TaiKang Center for Life and Medical Sciences, Frontier Science Centre of Immunology and Metabolism, Wuhan University, Wuhan, Hubei 430072, China; The First Affiliated Hospital of USTC, MOE Key Laboratory for Membraneless Organelles and Cellular Dynamics, Division of Life Sciences and Medicine, University of Science and Technology of China, Hefei, Anhui 230001, China; Hubei Key Laboratory of Cell Homeostasis, College of Life Sciences, TaiKang Center for Life and Medical Sciences, Frontier Science Centre of Immunology and Metabolism, Wuhan University, Wuhan, Hubei 430072, China; Hubei Key Laboratory of Cell Homeostasis, College of Life Sciences, TaiKang Center for Life and Medical Sciences, Frontier Science Centre of Immunology and Metabolism, Wuhan University, Wuhan, Hubei 430072, China; Hubei Key Laboratory of Cell Homeostasis, College of Life Sciences, TaiKang Center for Life and Medical Sciences, Frontier Science Centre of Immunology and Metabolism, Wuhan University, Wuhan, Hubei 430072, China; Hubei Key Laboratory of Cell Homeostasis, College of Life Sciences, TaiKang Center for Life and Medical Sciences, Frontier Science Centre of Immunology and Metabolism, Wuhan University, Wuhan, Hubei 430072, China; Hubei Key Laboratory of Cell Homeostasis, College of Life Sciences, TaiKang Center for Life and Medical Sciences, Frontier Science Centre of Immunology and Metabolism, Wuhan University, Wuhan, Hubei 430072, China; Department of Hematology, Nanjing Drum Tower Hospital, the Affiliated Hospital of Nanjing University Medical School, Nanjing, China; Hubei Key Laboratory of Cell Homeostasis, College of Life Sciences, TaiKang Center for Life and Medical Sciences, Frontier Science Centre of Immunology and Metabolism, Wuhan University, Wuhan, Hubei 430072, China; The First Affiliated Hospital of USTC, MOE Key Laboratory for Membraneless Organelles and Cellular Dynamics, Division of Life Sciences and Medicine, University of Science and Technology of China, Hefei, Anhui 230001, China; Beijing Key Laboratory of DNA Damage Response and College of Life Sciences, Capital Normal University, Beijing 100048, China; The MOE Key Laboratory of Biosystems Homeostasis & Protection, Zhejiang Provincial Key Laboratory for Cancer Molecular Cell Biology and Innovation Center for Cell Signaling Network, Life Sciences Institute, Zhejiang University, Hangzhou 310058, China; Hubei Key Laboratory of Cell Homeostasis, College of Life Sciences, TaiKang Center for Life and Medical Sciences, Frontier Science Centre of Immunology and Metabolism, Wuhan University, Wuhan, Hubei 430072, China

## Abstract

The single-stranded DNA (ssDNA) binding protein complex RPA plays a critical role in promoting DNA replication and multiple DNA repair pathways. However, how RPA is regulated to achieve its functions precisely in these processes remains elusive. Here, we found that proper acetylation and deacetylation of RPA are required to regulate RPA function in promoting high-fidelity DNA replication and repair. We show that yeast RPA is acetylated on multiple conserved lysines by the acetyltransferase NuA4 upon DNA damage. Mimicking constitutive RPA acetylation or blocking its acetylation causes spontaneous mutations with the signature of micro-homology-mediated large deletions or insertions. In parallel, improper RPA acetylation/deacetylation impairs DNA double-strand break (DSB) repair by the accurate gene conversion or break-induced replication while increasing the error-prone repair by single-strand annealing or alternative end joining. Mechanistically, we show that proper acetylation and deacetylation of RPA ensure its normal nuclear localization and ssDNA binding ability. Importantly, mutation of the equivalent residues in human RPA1 also impairs RPA binding on ssDNA, leading to attenuated RAD51 loading and homologous recombination repair. Thus, timely RPA acetylation and deacetylation likely represent a conserved mechanism promoting high-fidelity replication and repair while discriminating the error-prone repair mechanisms in eukaryotes.

## Graphical Abstract

**Graphical Abstract:**
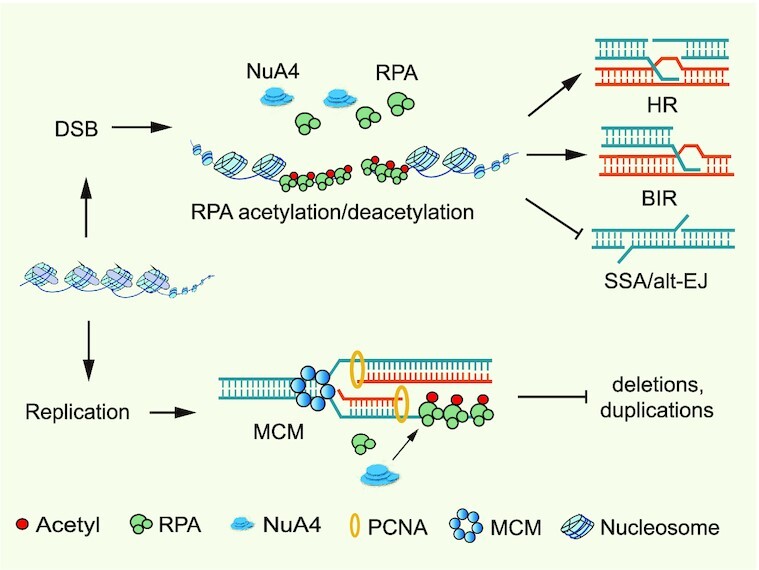
Proper RPA acetylation/deacetylation facilitates efficient or dynamic RPA binding on ssDNA. As a result, proper RPA acetylation favours the accurate DSB repair by gene conversion or BIR while suppressing spontaneous mutations and the error-prone alt-EJ or SSA repair mechanism.

## INTRODUCTION

Accumulation of errors during DNA replication and repair can lead to genome instability and cancer (1). Indeed, cancer cells often have complex genome rearrangements and mutation signatures, resulting from defects in different DNA repair or replication proteins (2–6). Therefore, the suppression of mutations and low-fidelity repair is critical to avoiding cancer and other diseases.

Single-stranded DNA (ssDNA) is one of the most ubiquitous and essential biological intermediates formed throughout DNA metabolism(7–9). However, exposed ssDNA can form secondary structures, impeding normal DNA transactions. In addition, ssDNA can be attacked by nucleases. Thus, exposed ssDNA poses a potential threat to genome stability. Replication Protein A (RPA), the principal ssDNA-binding protein complex in eukaryotes, binds ssDNA with a very high affinity (*K*d∼ 10^−9^ - 10^−11^ M) and is considered the first responder of ssDNA (7,10). RPA protects ssDNA from unscheduled nuclease digestion, melts secondary structures, and senses ssDNA to elicit checkpoint signals (7,8,11,12). It also serves as a key scaffold to recruit factors involved in different DNA transactions(7,8,11,13). As a result, RPA is critical for fundamental nuclear processes, including transcription, replication, repair, recombination, and chromosome segregation (14–19). While RPA is an essential protein, its dysfunction can cause mutations, genome instability, and chromosome catastrophe and is closely related to carcinogenesis (7–9,11,13,20–23).

RPA is a heteromeric complex composed of three subunits (Rfa1, Rfa2 and Rfa3). RPA contains a total of six oligonucleotide/oligosaccharide-binding motifs (OB), four in the Rfa1 subunit (OB-A, OB-B, OB-C and OB-F), and one each in Rfa2 (OB-D) and Rfa3 (OB-E)(7,8). Four of the domains primarily coordinate ssDNA interactions and are termed DNA binding domains (DBDs; DBD-A, B, C and D). These motifs mediate the dynamic association of RPA with ssDNA or proteins (7,11,24). RPA can bind short (8–10 nt) or long (28–30nt) ssDNA in different modes and diffuse on the bound DNA ligand (25–31). The cellular functions of RPA rely on its high ssDNA-binding affinity and its ability to interact with different proteins (25). Although RPA has a high affinity for ssDNA, recent studies have suggested that the binding of RPA on ssDNA requires additional regulations(32–34).

RPA plays an indispensable role in DNA replication and repair. During DNA replication, RPA is required for both replication initiation and elongation(15,17,18). In addition, RPA facilitates polymerase loading and replication-coupled nucleosome assembly (17,18,35–39). RPA is also critical for homologous recombination (HR), an essential mechanism for the repair of DNA double-strand breaks (DSB) or the restart of stalled or collapsed replication forks (40–44). During HR repair, the 5′-ssDNA of DSB ends are selectively degraded by resection machinery, generating 3′-ssDNA (45,46). RPA rapidly coats the exposed 3′-ssDNA and elicits signals to activate the DNA damage checkpoint. The recombinase Rad51 subsequently replaces RPA to form the Rad51-ssDNA presynaptic filament. The Rad51-ssDNA filament drives the invasion of the 3′-strand at the homologous sequence to form the D-loop structure, followed by repair DNA synthesis and resolution of recombination intermediates(40–44). A long-standing question is how RPA is precisely regulated to ensure high-fidelity replication and repair.

Post-translational modifications of RPA exert essential roles in preserving genome integrity. For example, the phosphorylation or sumoylation of RPA regulates DNA replication, damage response, HR repair, checkpoint signaling, and RPA interaction with its partners (8,47–51). Meanwhile, the ubiquitination of RPA by the E3 ligase RFWD3 facilitates HR repair at stalled forks or interstrand crosslink sites, likely via facilitating the degradation or removal of RPA and RAD51 from the lesion sites(52,53). Notably, recent studies showed that RPA is acetylated in both yeast and human upon DNA damage(54–56). In yeast, RPA was reported to be acetylated by the acetyltransferase NuA4 on lysines K259, K427, K463 and K494 of Rfa1 in response to MMS treatment (54). This acetylation appears to attenuate RPA binding on ssDNA and is required for the DNA damage response (54). In parallel, human RPA1 is acetylated on K163 by the acetyltransferases GCN5 and PCAF, and this acetylation promotes nucleotide excision repair of UV-induced DNA damage(55,56). However, whether RPA acetylation affects the repair of DSBs, the most deleterious DNA lesions, remains to be determined.

In this study, we investigated the impact of RPA acetylation and deacetylation on spontaneous mutations and DSB repair. We show that proper acetylation and deacetylation of RPA promote high-fidelity DNA replication and recombination while suppressing mutations and low-fidelity repair pathways. We also provide evidence that this regulation plays a similar role in human cells. Thus, our study reveals a conserved mechanism by which eukaryotic cells ensure the fidelity of replication or repair and extends the understanding of the role of RPA post-translational modifications.

## MATERIALS AND METHODS

### Yeast strains and cell culture

Yeast strains are listed in Supplementary Table S1. HEK293T and HeLa cells were cultured in Dulbecco′s modified essential medium containing 10% fetal bovine serum with 100 units/ml penicillin and 100 μg/ml streptomycin. U2OS cells were cultured in 20% fetal bovine serum, and all cells were maintained in an atmosphere containing 5% CO_2_ at 37°C.

### DNA damage sensitivity test

Yeast cells were cultured in the YPD medium (1% yeast extract, 2% peptone and 2% dextrose) overnight to saturation. Undiluted cell culture and 1/10 serial dilutions of cell cultures were spotted onto YPD plates containing indicated concentrations of camptothecin, phleomycin, zeocin, or MMS. Plates were incubated at 30°C for 2–3 days before taking pictures.

### Fluorescence microscopy

Yeast strains carrying the RFA1-YFP and Nup49-mCherry fusion proteins were grown at 30°C to mid-log before being harvested. Cells were resuspended in 1.7 μl sterile deionized water on the glass slide and examined using a ZEISS LSM 880 fluorescence confocal microscope. Images were analyzed by Zen Application Service. Approximately 100 cells were counted for each experiment.

### Mutation rate and spectra

The rate of accumulation of CanR mutations was determined as previously described (57). Yeast cells from single fresh colonies were plated on SC arginine-dropout plates containing 60 mg/L canavanine. The mutation rate was measured by fluctuation analysis using the median method. CanR mutation spectra were characterized by PCR amplification of the *CAN1* gene from independent CanR isolates, followed by DNA sequencing.

### Yeast gene conversion, single-strand annealing, and alt-EJ assays

To test the viability of DSB repair by gene conversion (tGI354), SSA (yWH378) or alt-EJ (JKM139), we cultured cells in the pre-induction medium (YEP-Raffinose) overnight to the log phase. Cells were diluted and plated on YEPD and YEP-Gal plates, respectively, then incubated at 30°C for 3–5 days. The survival rate was calculated by dividing the number of colonies grown on YEP-Gal by the number of colonies on YEPD (x100%). While the measurement of DNA end resection or repair kinetics for ectopic recombination by Southern blot was performed as described(58).

To measure the cell survival in the NA14 system, we cultured cells in the pre-induction medium YEP-Raffinose overnight to the log phase. Next, 2% of galactose was added to the medium, and cells were allowed to continue growing for additional 10 h. Cells were then washed and plated on YEPD and incubated at 30°C for three days. Colonies were then analyzed by replica plating to selective media containing G418 (300 mg/ml). At least three independent experiments were performed for each strain.

### Yeast BIR assay

Allelic BIR assay was performed as previously described (59,60). The frequencies of BIR, gene conversion, half crossovers, and chromosome loss were measured based on the percentage of colonies carrying markers specific to these repair outcomes, as reported previously (59,60). The repair efficiency was calculated as the percentage of normalized pixel intensity of the BIR product band compared to the normalized parental bands at 0 h. Quantitative analysis was completed with ImageQuant TL 5.2 software (GE Healthcare Life Sciences).

### Chromatin immunoprecipitation (ChIP)

Log phase yeast cells (∼1 × 10^7^ cells/ml) grown in YEP-Raffinose medium were induced with 2% galactose to generate DSBs. Samples were collected at 0 or 4hr after DSB induction. Chromatin DNA was sheared to an average size of ∼300 bp using a Diagenode Bioruptor. ChIP and qPCR assays were carried out as previously described(34). The anti-FLAG and anti-Myc antibodies used for ChIP were ordered from CST (#20E3) and MBL(M192- (3), respectively.

### Recombination protein expression and purification

The WT, 4KQ or 4KR *RFA1* allele was individually cloned into the vector pGEX-4T-3 and transformed into BL21(DE3) *E. coli* cells. When the culture was grown to an OD_600_ of 0.4–0.6, 0.1mM IPTG was added to induce protein expression. Cells were cultured at 16°C for 16 h before harvest. The cell pellet was resuspended in 1xPBS, followed by lysing with sonication. After centrifugation at 16 000 g for 15 min, the supernatant was collected and filtered through a 0.45 μm sterile syringe filter. The supernatant was then mixed gently with GST agarose beads at 4°C for 2 h with agitation. The GST agarose was washed extensively with the purification buffer (50 mM Tris–HCl, pH 7.5, 100 mM NaCl, 5 mM EDTA, 1% Triton X-100), followed by elution with 20 mM reduced glutathione. The purification of the NuA4 complex was carried out as described by Wang et al. (61).

### Immunoprecipitation and Western blot

Yeast cells were grown at 30°C overnight to log phase. Cells were treated with or without 0.1% MMS for 1 h before harvest. Cells were then resuspended in IP lysis buffer (100 mM HEPES, 100 mM KAC, 2 mM MgCl_2_, 2 mM Beta-ME, 0.1% NP-40, 1 mM PMSF, 1× protease Inhibitor Cocktail, 5 mM Na Butyrate, 5 mM Nicotinamide, and 1 μg/ml TSA) and mechanically disrupted using glass beads at 4°C. The lysates were collected and digested with UltraNuclease (YEASEN Biotech) at 37°C for 20 min to solubilize chromatin-bound proteins. For immunoprecipitation, each sample was incubated with 10 μl of anti-acetyl-lysine (Immunechem, ICP0380) or 3 μl of anti-FLAG (MBL, M185-3L) or anti-HA (MBL, M180-3) antibody for 4 h or overnight at 4°C. Afterward, the mixture was added with protein G-sepharose and incubated at 4°C for 3 h with agitation. The beads were washed with the IP lysis buffer supplemented with 140 mM NaCl four times and resuspended in 50 μl of 2× SDS loading buffer. Immunoprecipitated proteins were analyzed by Western blot using an anti-FLAG antibody (Sigma, F1804).

For immunoprecipitations in human cells, HEK293T cells were washed with 1× PBS and lysed in the NETN buffer (20 mM Tris–HCl, pH 8.0, 1 mM EDTA, 100 mM NaCl, 0.5% NP-40, supplemented with protease inhibitors) containing deacetylase inhibitors (5 mM nicotinamide and 1 μg/ml TSA) for 30 min on the ice. After centrifugation at 16 000 g for 15 min, the supernatant was collected and precleared with the protein G-agarose beads (GE Healthcare). The beads were removed by centrifugation, and the supernatant was incubated with an anti-acetyl lysine antibody (Immunechem, ICP0380) at 4°C for 6 h. Next, 30 μl of protein G-agarose beads were added to each reaction, and the mixture was incubated at 4°C with agitation for additional 3 h. Finally, the beads were washed extensively with the NETN buffer supplemented with deacetylase inhibitors, followed by boiling in 2× SDS loading buffer.

Products from immunoprecipitation, pull down or the whole cell lysates were resolved on an 8.5% SDS-PAGE followed by transferring onto a PVDF (Immobilon-P, Millipore) membrane using the semi-dry method (Bio-Rad). For Western blot analysis, the anti-FLAG (F3165 or F1804) and anti-HA (30701ES60) antibodies were purchased from Sigma and YESEN, respectively. The anti-GAPDH and anti-GST(AE001) antibodies were purchased from Abclonal. The anti-His (No. 66005-1-Ig) antibody was ordered from Proteintech. The human RPA1(ab176467) antibody was purchased from Abcam. The anti-mouse and rabbit IgG HRP-conjugated secondary antibodies were purchased from Santa Cruz Biotechnology. Blots were developed using the western blotting substrate (Bio-Rad).

### *In vitro* acetylation assays

*In vitro* acetylation assay was performed as described (54). Briefly, the acetylation was carried out in a 15 μl of reaction system containing 10 nM of the NuA4 complex, 100 nM of the GST-RPA complex (or 40 nM of GST-RFA1 or GST-RFA1-4KR), 0.25 μCi of ^3^H-acetyl CoA (Perkin Elmer), 10 mM Na Butyrate, 25 mM KCl, and 3 μl of 5× HAT buffer (250 mM Tris pH 8.0, 25% glycerol, 0.5 mM EDTA, 5 mM DTT, 5 mM PMSF). The reaction was incubated at 30°C for 30 min. The reaction product was spotted on a PVDF membrane for the liquid assay. After air drying, the membrane was washed three times with 50 mM carbonate buffer (0.5 M Na_2_CO_3-_NaHCO_3_, pH 9.2), followed by rinsing with acetone. The membrane was then placed in a scintillation vial, followed by the addition of a scintillation cocktail, and the radioactive signals were counted by a liquid scintillation analyzer (Tri-carb 2910TR, PerkinElmer).

### Biotin-ssDNA pull-down assay

The 5′-biotinylated ssDNA (30 nt, 5′-cgataagcttgatatcgaattcctgcagcc-3′) was immobilized to streptavidin-coated magnetic beads (GenScript) by incubating at room temperature for 20 min in 1×PBS containing 0.01% Triton X-100 and 1 mM EDTA. After washing with 1×PBS, the beads were resuspended with 15 μl of reaction buffer (250 mM Tris–HCl, pH 8.0, 25% glycerol, 0.5 mM EDTA, 5 mM DTT, 10 mM Na Butyrate, 25 mM KCl, 5 mM PMSF). Then, 10 nM of NuA4, 40 nM of GST-RFA1, and 0.25 μCi of ^3^H-acetyl CoA were added sequentially to initiate the acetylation reaction, and the mixture was incubated at 30°C for 1 hr. The beads were washed with washing buffer (50 mM Tris–HCl, pH 7.5, 1 mM DTT, 1 mM PMSF, 10 mM Na-butyrate, 0.5 mM MgCl_2_, 40 mM KCl) three times and resuspended in 2×loading buffer. The ssDNA-bound RPA was eluted by boiling the beads in the loading buffer.

To compare the ssDNA binding ability of human RPA1-WT, 3KQ and 3KR mutant protein, HEK293T cells were transfected with the pCDNA5.0 plasmid expressing the FLAG-tagged RPA1-WT, 3KQ or 3KR using the Gene Twin transfection reagent. After 24 h, cells were washed with 1xPBS three times and harvested, and then lysed in the NETN lysis buffer (20 mM Tris–HCl, pH 8.0, 1 mM EDTA, and 0.5% NP-40, 100 mM NaCl, supplemented with protease inhibitors) on ice for 30 min. After centrifugation, the supernatant was collected and incubated with the biotin-ssDNA-streptavidin beads for 30 min at room temperature. Beads were washed with the NETN buffer three times, followed by boiling in 2×SDS loading buffer. The eluted proteins were analyzed by Western blot. Analysis of the ssDNA binding ability of yeast RFA1-WT, 4KQ, or 4KR was conducted as described above, except that the lysis buffer and wash buffer used were the same as the ones used for acetyl-lysine immunoprecipitation in yeast.

### Electrophoretic mobility shift assay (EMSA)

20 nM of 5′-Cy3 labeled ssDNA (30 or 90 nt) was incubated with indicated amounts of the WT, 4KQ or 4KR RPA complex (10, 20 or 30 nM) at room temperature for 30 min in 50 mM Tris–HCl, pH 8.0. The reaction mixture (10 μl) was mixed with 3 μl of 6×DNA loading dye and loaded onto a 6% native bis-acrylamide gel and resolved using cold 0.3×TBE buffer for 20 min. The gel was scanned using Typhoon FLA 9500 imager (GE Healthcare), and band intensities were quantified using the ImageJ software.

### Single-molecule study

Single-molecule studies monitoring RPA binding on ssDNA were performed as previously described (34). The 12.5 k-nt ssDNA was produced by one-sided PCR, and its two ends were labeled with digoxigenin and biotin groups, respectively. First, the digoxigenin-labeled end of a single ssDNA molecule was anchored to the anti-digoxigenin-coated glass surface in a flow cell. Then, the biotin-labeled end of the anchored ssDNA molecule was attached to a superparamagnetic microbead (M-270, Dynal beads). A pair of permanent magnets were used to attract the microbead and thus exert a constant force on the anchored ssDNA molecule. The extension of ssDNA was determined to be the separation between the microbead and glass surface. The assembling buffer contained 100 mM NaAc, 10 mM MgAc_2_, 1 mM ATP and 25 mM Tris-Ac, pH 7.5. All experiments were performed at a constant force of 4.3 pN at 21°C.

### Microscale thermophoresis assay

Purified recombinant 6xHis-Rtt105 protein was labeled with a RED-tris-NTA protein labelling kit (Nano Temper) following the manufacturer's instruction. The labeled protein was incubated at a constant concentration (100 nM) with serial dilutions of the WT, 4KQ, or 4KR RPA complex (from 200 to 0.0058 nM) in the MST buffer (1× PBS with 0.05% Tween 20). Equal volumes of proteins were mixed by pipetting and incubated for 50 min at room temperature. The reaction mixtures were enclosed in Monolith™ NT.115 Series Capillaries and loaded into the instrument (Monolith NT.115, Nano Temper, Germany). The measurement procedures and *K*_d_ value analysis were determined using the Nano Temper analysis tool.

### Human HR, BIR, alt-EJ and SSA reporter assays

HR, BIR, alt-EJ and SSA were measured using the reporter assays following the previously described procedures. The DR-GFP (62) and alt-EJ-EGFP (63) reporters in U2OS cells were gifted by Dr Xingzhi Xu (Shenzhen University), and the EGFP-BIR-5085 (64) and SSA-EGFP (65) reporters in U2OS cells were provided by Dr Hailong Wang (Capital Normal University) and Dr Jun Huang (Zhejiang University), respectively. The siRNA transfection was performed with 100 pm of RPA1 siRNA duplexes using Lipofectamine 2000 reagent following the manufacturer's instruction. Four hours after siRNA transfection, a plasmid expressing the FLAG-tagged siRNA-resistant *RPA1*-WT, *-3KQ* or *-3KR* allele was transfected into the reporter system. For analyzing HR, BIR or SSA, cells transfected with indicated plasmids were then transiently transfected with the I-*Sce*I expressing vector pCBAScel (Addgene). After 48 h, cells were harvested, and the expression of EGFP was analyzed by flow cytometry. For alt-EJ, cells transfected with indicated plasmids were then supplemented with 10 μg/ml doxycycline to induce DSBs. After 24 h, the percentage of GFP-positive cells was analyzed by flow cytometry. The relative efficiency of HR, BIR, alt-EJ or SSA was normalized to that of control cells. At least 10000 cells were counted for each sample. The values presented are the analysis of three independent experiments.

### Immunofluorescence staining

HeLa cells were seeded on coverslips in 12 wells plates 24 h before experiments. For staining γH2AX foci, cells were treated with HU (5 mM) for 4 h and then allowed to recover for 10–24 h. For staining RPA and RAD51 foci, cells were treated with VP16 (5 μM) for 1 h before sampling. Cells on coverslips were washed with 1×PBS, then fixed with 4% paraformaldehyde for 15 min at room temperature and permeabilized with 0.5% Triton X-100 for an extra 15 min. After being blocked with 5% BSA, cells on coverslips were incubated with the anti-γH2AX (ab26350, Abcam), anti-RPA1(ab176467, Abcam) or anti-RAD51(ab133534, Abcam) antibody at 4°C overnight. Following washing with PBS three times, secondary antibodies were added and incubated at room temperature for 1 h. Cells were then stained with DAPI to visualize nuclear DNA. Images were captured using Leica SP8 inverted fluorescent microscope with a 63×objective and processed using Leica Application Suite X software. Quantification of the signals was carried out using ImageJ. Statistical analysis was performed using Prism (GraphPad Software). Statistical significance was determined by the two-tailed *t*-test.

### Cell viability assay

Cell viability was measured using the CCK-8 assay (YEASEN Biotech). HeLa cells stably expressing the FLAG-tagged WT, *3KQ* or *3KR RPA*1 allele (siRNA resistant) were seeded at a density of 5 × 10^3^ cells/well in 96-well plates. After 24hrs, cells were transfected with siRNA against RPA1 using the Lipofectamine 2000 reagent. Cells were then incubated for 24 h before treating with iniparib (MCE) at indicated doses (0, 10, 25, 50 and 100 μM). After 24hr treatment, 10 μl CCK-8 reagent was added to each well, and the plates were incubated for 1 h at 37°C. Finally, the absorbance was measured at 450 nm using a scanning microplate reader (Cytation3). Cell viability at individual time points was normalized to the untreated group.

## RESULTS

### RPA is acetylated in vivo and in vitro by NuA4

To characterize the role of RPA acetylation in preserving genome stability, we first immunoprecipitated acetylated proteins from yeast lysates with a pan-anti-acetyl-lysine antibody and tested RPA acetylation by immunoblotting with an anti-FLAG antibody. In line with previous studies, we noted that yeast RPA was acetylated at a low level in unperturbed conditions, and the acetylation was enhanced upon methyl methane sulfonate (MMS) treatment that can induce DSBs (Supplementary Figure S1A) (54). Furthermore, we observed that RPA acetylation was dependent on the acetyl-transferase NuA4 since the depletion of Esa1, the catalytic subunit of the NuA4 complex, by shifting the *esa1-L254P* temperature-sensitive mutant to a non-permissive temperature, significantly impaired RPA acetylation (Supplementary Figure S1B), as previously noted (54). Consistently, purified yeast NuA4 complex directly acetylated the recombinant RPA complex *in vitro*, as measured by a liquid scintillation spectrometer (Supplementary Figure S1C–E). Previous work by Cheng et al. (2018) identified four acetylation sites (lysine K259, K427, K463 and K494) on yeast Rfa1 using mass spectrometry analysis (54). We noted that three of the four lysines are conserved across species and located on surfaces of the core DBD structures (Supplementary Figures S1F and G). Indeed, simultaneous mutation of the four lysines on Rfa1 to arginine greatly attenuated RPA acetylation *in vitro* and *in vivo* (Supplementary Figures S1H and I). Consistently, we did not detect noticeable acetylation on the purified GST-Rfa2 or GST-Rfa3 subunit by NuA4 *in vitro* (Supplementary Figure S2). Thus, the RPA complex is acetylated by NuA4 primarily on Rfa1, and this acetylation could be stimulated by DNA damage.

### Proper acetylation and deacetylation of RPA promotes DNA damage response and repair

**Figure 1.**
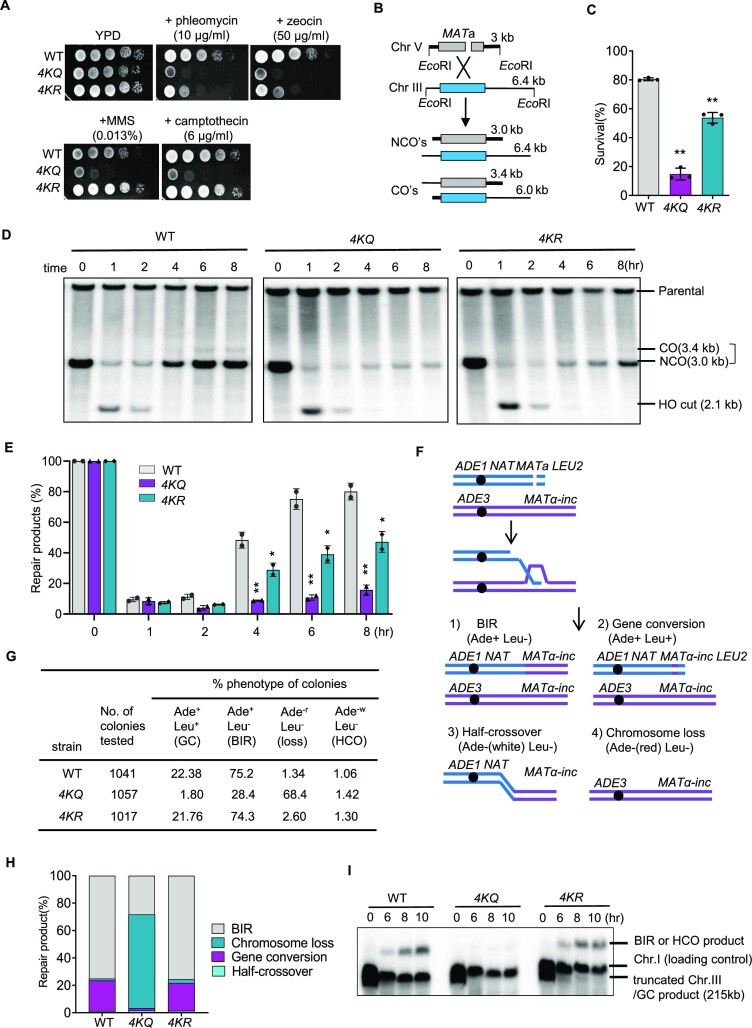
Proper RPA acetylation and deacetylation promote DSB repair by gene conversion or BIR. (**A**) Sensitivity test for the WT, *4KQ* or *4KR* cells in response to different DNA damaging agents at indicated concentrations. Cells were grown on YPD (1% yeast extract, 2% peptone, and 2% dextrose) plates. (**B**) Scheme showing an ectopic recombination system. CO: crossover; NCO: non-crossover. (**C**) The survival rate for indicated yeast cells repaired by ectopic recombination. (**D**–**E**) Southern blot analysis and quantification of the repair kinetics for the ectopic recombination. The blot was hybridized with the *MAT*a probe. The repair products in (**D**) are indicated by the bracket. (**F**) Diagram showing a site-specific BIR system. Repair of DSBs by different mechanisms generates distinct repair products that can be determined by following the markers on the chromosomes. (**G**) Table showing the BIR repair outcomes in the WT, *4KQ* or *4KR* mutant cells. Cells cultured in the pre-induction liquid media were plated on YEP-Galactose media to induce DSBs. Colonies were then replica plated on Leu^−^ or Ade^−^ dropout media. The frequencies of BIR, half-crossover (HCO), gene conversion (GC), and chromosome loss(loss) were calculated based on the percentage of colonies carrying markers specific to these repair outcomes. (**H**) Graph showing the proportion of each category of repair products in indicated strains. (**I**) Southern blot analysis showing the repair kinetics of BIR for indicated strains. *ADE1* was used as a probe. Error bars in this figure represent the standard deviation from at least three independent experiments. Statistical analysis was calculated with the Student's *t*-test. **P < 0.05, **P< 0.01.

To test the role of RPA acetylation in the DNA damage response, we constructed the *rfa1-4KR* (*4KR*) and *rfa1-4KQ* (*4KQ*) point mutants wherein the four lysines (K259, K427, K463 and K494) were simultaneously mutated to arginine to block the acetylation or glutamine to mimic constitutive acetylation. Unlike wild-type (WT) cells, blocking RPA acetylation (*4KR*) caused hypersensitivity to phleomycin or zeocin, which can induce DSBs. However, mimicking constitutive RPA acetylation (*4KQ*) resulted in a more severe defect in response to all tested DNA-damaging agents, including phleomycin, zeocin, MMS and camptothecin (Figure 1A).

To determine which residue is the key acetylation site in cells, we mutated these sites individually and carried out immunoprecipitation with the pan-anti-acetyl-lysine antibody. The product was analyzed by immunoblotting with the anti-FLAG antibody. We noted that the K427R or K494R single-point mutation did not reduce RPA acetylation (Supplementary Figure S3A). However, K463R and, to a lesser extent, K259R mutation impaired RPA acetylation, and a combined mutation of the four sites (*4KR*) further reduced RPA acetylation. Notably, mutation of any of these single residues to glutamine or simultaneous mutation of K259 and K463 to glutamine caused no or only marginal defects in the DNA damage sensitivity, in contrast to that observed in the *4KQ* mutant (Supplementary Figure S3B). These results suggest that although K463 and K259 appear to be the key acetylation sites, the acetylation of RPA on the four residues functions collectively to promote the DNA damage response.

The DNA damage sensitivity in the *4KQ* or *4KR* mutant was not caused by any defects in the DNA damage checkpoint because both mutants can efficiently activate the checkpoint upon DSB induction, as reflected by the status of Rad53 phosphorylation and G2/M arrestment (Supplementary Figures S4A and B). Together, these results suggest that proper acetylation and deacetylation of RPA are required to stimulate DNA damage response or repair.

### Proper acetylation and deacetylation of RPA promotes DSB repair by gene conversion

Next, we tested the role of proper RPA acetylation in DSB repair by HR. We employed an ectopic recombination system wherein the HO endonuclease generates a single DSB at the *MAT***a** sequence inserted at the *ARG5,6* locus on chromosome V. The DSB is repaired by HR using the homologous *MAT***a**-inc sequence on chromosome III as a template (Figure 1B) (66). ∼80% of the WT cells completed the repair and survived, while the survival rate was reduced to 52% for the *4KR* mutant and 16% for the *4KQ* mutant (Figure 1C). Moreover, both mutants repaired the break with much slower kinetics, as revealed by the Southern blot analysis (Figures 1D and E). In contrast to the *4KQ* mutant, mutation of the four residues individually to glutamine has only modest or no defect in the HR repair (Supplementary Figure S3C), supporting the conclusion that RPA acetylation on these sites functions collectively. These results indicate that proper acetylation and deacetylation of RPA are important for DSB repair by gene conversion.

We then tested the effect of proper RPA acetylation on break-induced replication (BIR), a unique HR mechanism for repairing one-end DSBs that occur at collapsed replication forks or eroded telomeres(67). We used a BIR system in which only one end of the HO-induced DSB has extensive homology to the template sequence so that ∼70% of cells use BIR to copy over 100 kb of chromosome III to complete the repair (59,60). >20% of the remaining cells repaired the break via gene conversion by capturing the second end of the DSB. The repair outcome or chromosome loss was determined by following the genetic markers (Figure 1F) (59,60). Compared to the WT cells, the repair pattern or outcome in the *4KR* mutant remains largely unaffected, except that chromosome loss was slightly increased (Figures 1G and H). However, the repair outcome is markedly changed in the *4KQ* mutant, with gene conversion reduced from 22% to 1.8% and BIR reduced from 75% to 28%, accompanied by a massive chromosome loss (from 1.34% to 68.4%, ∼50-fold) (Figures 1G and H). Consistently, we failed to detect BIR products within 10 hours following DSB induction in the *4KQ* mutant cells (Figure 1I). Notably, we observed frequent chromosome rearrangements in this mutant (Supplementary Figures S5A-C). Thus, timely deacetylation of RPA is crucial to promote DSB repair by gene conversion or BIR while preventing chromosome loss. These results establish an important role of proper RPA acetylation and deacetylation in the repair of DSBs by HR.

### Proper RPA acetylation favors DSB repair by the accurate HR while discriminating the error-prone pathways

**Figure 2.**
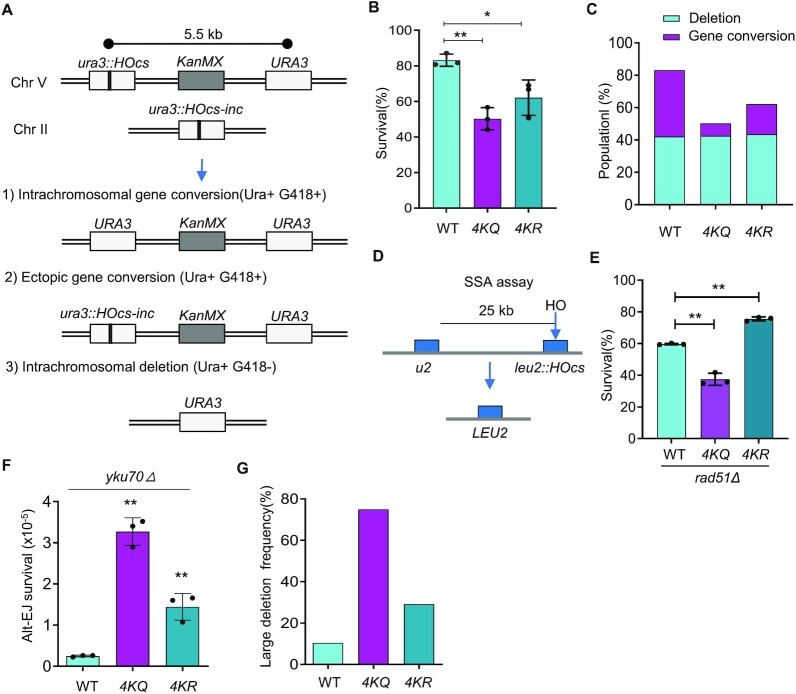
Proper RPA acetylation and deacetylation promote high-fidelity HR repair while suppressing the deleterious SSA or alt-EJ pathway. (**A**) Scheme showing a DSB repair system. A single DSB is generated on chromosome V upon the induction of the HO endonuclease by galactose. The DSB is primarily repaired by gene conversion using an intrachromosomal or ectopic template or by SSA that leads to deletions. The repair by gene conversion or SSA can be distinguished by following the marker on chromosomes. (**B**) The survival rate of DSB repair by gene conversion or SSA for indicated strains. (**C**) Graph showing the proportion of repair products for gene conversion and SSA in indicated strains. (**D**) Scheme showing an SSA repair system. Two partial *leu2* repeats separated by a 25-kb interval sequence can anneal via SSA to repair the HO-induced DSB. (**E**) The survival rate for SSA in indicated cells. (**F**) The survival rate for DSB repaired by alt-EJ in indicated cells. (**G**) Graph showing the proportion of repair products with large deletions in indicated strains repaired by alt-EJ. Error bars represent the standard deviation from at least three independent experiments. Statistical analysis was calculated with the Student's *t*-test. *P < 0.05, **P < 0.01.

We then asked whether RPA acetylation affects the choice of DSB repair pathways. We employed a haploid yeast strain (NA29) wherein a single HO-induced DSB is repaired primarily by the accurate intrachromosomal or ectopic recombination or by the deleterious intrachromosomal single-strand annealing (SSA) that anneals the complementary ssDNA revealed by resection and leads to the deletion of the intervening sequence (Figure 2A) (68,69). In this system, two copies of the *URA3* genes (direct repeats) sharing 1.2-kb homology were inserted on chromosome V, separated by a 5.5 kb interval (Figure 2A). One of them carries a HO cut site, while the second copy is a WT *URA3* gene. A third 1.2-kb fragment of the *URA3* gene with a mutated HO recognition site (*MAT*a-inc) is present at the *LYS2* locus on chromosome II. A single DSB can be induced by the HO endonuclease upon galactose induction. By following the genetic markers, we could distinguish the products repaired by gene conversion (Ura + G418^R^) or SSA (Ura + G418^S^). 83% of WT cells completed the DSB repair and survived, while the survival rate was reduced to 50% for the *4KQ* mutant and ∼60% for the *4KR* mutant (Figure 2B). As reported, approximately half of the survivors for WT cells were repaired by gene conversion, while the other half were repaired by SSA (deletions) (68). Notably, the repair by gene conversion was reduced to 7.6% in the *4KQ* mutant and 18.5% in the *4KR* cells, while the repair by SSA appeared to remain unaffected in both mutants (Figure 2C). This was surprising because RPA was known to suppress the Rad52-mediated annealing of complementary ssDNA (7,29,34). We reasoned that the effect of RPA acetylation on SSA might be minimized by the presence of ectopic recombination in this system.

To directly measure the impact of RPA acetylation on SSA repair, we employed an SSA assay wherein two partial *leu2* repeats separated by 25-kb interval on chromosome III can anneal to complete the HO-induced DSB repair (Figure 2D) (70,71). Rad51, which is required for typical HR but is dispensable for SSA, was deleted so that SSA is the only pathway to repair the DSB. Approximately 60% of WT cells survived, while ∼76% of *4KR* cells successfully completed the repair (Figure 2E), suggesting that proper RPA acetylation is required to suppress excessive SSA repair. This is in line with the role of RPA in restraining SSA (29,34). In contrast, we noted that the survival rate was reduced to 37% in the *4KQ* mutant (Figure 2E), implying that constitutive RPA acetylation might affect additional steps required for SSA repair.

The binding of RPA on ssDNA suppresses DSB repair by alternative end joining (alt-EJ), a Ku-independent error-prone process that requires the annealing of short homologies (29,72,73). Therefore, we tested the effect of RPA acetylation on alt-EJ in the *YKU70*-deleted JKM139 strain, wherein the HR pathway is blocked so that the DSB can only be repaired by alt-EJ. Notably, the alt-EJ rate is increased by 5-fold in the *4KR* mutant and by 12-fold in the *4KQ* mutant (Figure 2F). In addition, we observed that ∼10% of repair products in the WT cells harbor large deletions at the breakpoint, and the proportion was increased to 30% for *4KR* and 75% for *4KQ* mutant cells (Figure 2G and Supplementary Figure S6). Together, these results suggest that proper RPA acetylation and deacetylation facilitate the accurate repair by gene conversion while discriminating the aberrant repair by SSA or alt-EJ that leads to deletions.

### Proper RPA acetylation and deacetylation are required to suppress spontaneous large deletions, duplications and chromosome loss

**Figure 3.**
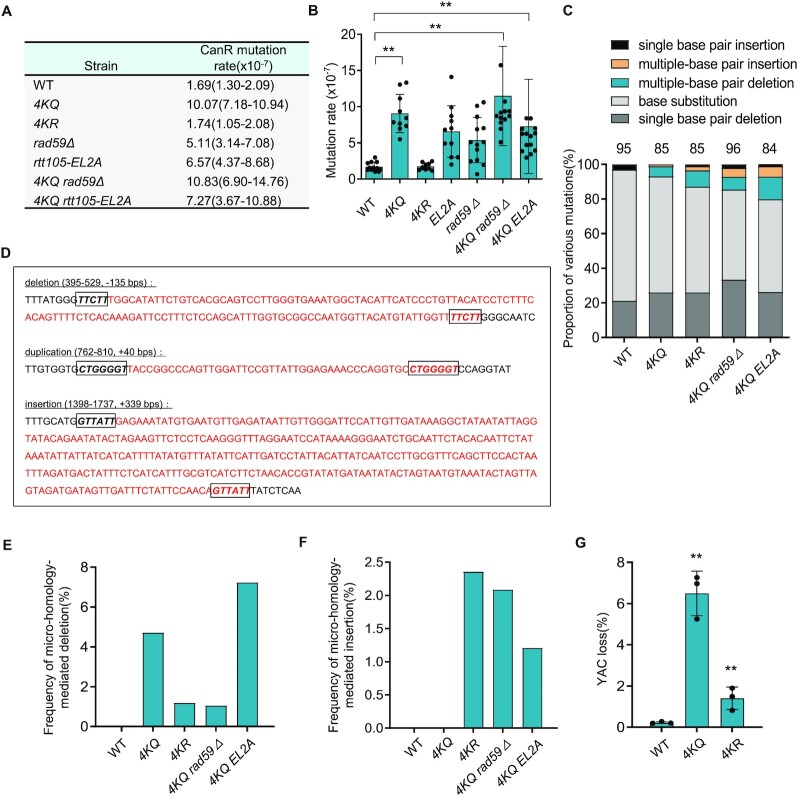
Proper RPA acetylation promotes high-fidelity DNA replication and suppresses chromosome loss. (**A**)Table listing the mutation rates for indicated strains. The values in the brackets represent 95% confidence intervals. (**B**) Plots showing the rate of spontaneous mutations in indicated cells. (**C**) Graph showing the proportion of each category of mutation in CanR colonies derived from indicated cells. The number of CanR colonies sequenced for each strain is indicated. (**D**) Examples of large deletions or duplications observed in the *4KQ* or *4KR* mutant cells. The deleted or duplicated DNA sequences are marked in red. The boxed sequences in italics denote micro-homologies. (**E**, **F**) Plots showing the frequency of large deletions and duplications, respectively, in indicated strains. (**G**) The frequency of YAC loss in the WT and mutant cells. Error bars in (B) and (G) represent the standard deviation from at least three independent experiments. **P< 0.01 (Student's *t*-test).

One of the key functions of RPA is to suppress mutations or chromosome catastrophe (7,11,13). We used the *CAN1* gene as a mutation reporter to test whether proper RPA acetylation affects the fidelity of DNA replication (57). Mutations in the *CAN1* gene generate canavanine-resistant (CanR) colonies that can be screened on SC arginine-dropout plates containing canavanine. In the WT cells, the spontaneous mutation rate is about 1.7 × 10^−7^. The mutation rate remained unchanged in *4KR* cells, while it was increased to ∼10 × 10^−7^ (∼5-fold) in *4KQ* cells (Figures 3A and B). To identify the nature of the mutations, we sequenced the *CAN1* gene from individual CanR colonies derived from the WT or mutant cells. We found that ∼ 77% of the mutations in WT cells were base substitutions, while the remaining were single base pair deletions (∼20%) or single base pair insertions (∼3%) (Figure 3C and Supplementary Figure S7). The mutation pattern is altered in the two mutants. Specifically, we observed ∼6% of multi-base pair deletions with sizes ranging from 2 to 135 bps in the *4KQ* mutant cells and 9% of such deletions in the *4KR* mutant (Figure 3C and Supplementary Figure S7). Notably, 80% of the multi-base pair deletions in the *4KQ* mutant are large deletions occurring between direct repeats with short micro-homologies, with sizes ranging from 63 to 135 bp. In contrast, only 12.5% of multi-base pair deletions in the *4KR* mutant belong to micro-homology-mediated large deletion events, while the rest are small deletions (Figures 3D and E and Supplementary Figures S7–S9). We also detected two large micro-homology-mediated insertion events in the *4KR* mutant cells. Both insertions are tandem duplications of DNA fragments with sizes ranging from 23 to 49 bps (Figures 3D and F and Supplementary Figures S7–S9). Notably, these gross deletions or duplications did not occur in WT cells (Figure 3E and F and Supplementary Figure S7) (34,74). These results suggest that proper acetylation and deacetylation of RPA suppress mutations with the signature of micro-homology-mediated large deletions or insertions.

Micro-homology-mediated deletions or duplications were reported to associate with polymerase slippage or SSA (74–79). Indeed, additional deletion of *RAD59*, which is required for SSA (80,81), in the *4KQ* mutant greatly reduced the micro-homology-mediated large deletions, yet it led to an increase of micro-homology-mediated large insertions (Figures 3E and F). Interestingly, we observed two unique large insertion events in the *4KQ rad59Δ* double mutant (Supplementary Figures S7–S8), i.e. an insertion of a 329-bp Ty1 retrotransposon element YPRCdelta18 at +1398 bp and an insertion of a 342-bp of YCLWdelta15 fragment at +149 bp position. How retrotransposon elements were activated in this mutant remains to be determined.

Finally, we evaluated the effect of the RPA mutations on chromosome loss using a system that carries an extra ∼320-kb yeast artificial chromosome (YAC) (82). We observed that the *4KQ* and *4KR* mutations led to a 30-fold and 6-fold increase, respectively, in YAC loss compared to the WT cells (Figure 3G). Together, these results indicate that proper acetylation and deacetylation of RPA are important for suppressing chromosome loss and spontaneous mutations with the signature of micro-homology-mediated large insertions or deletions.

### Proper RPA acetylation and deacetylation facilitate the loading of RPA, Rad52 and Rad51 at DSB ends

To explore how proper RPA acetylation may impact HR repair, we first measured DNA end resection using a system wherein a single HO-cut is induced on the *MAT*a locus on chromosome III upon the addition of galactose(70). The homologous sequence *HML* and *HMR* were deleted to prevent the repair by HR so that resection could proceed persistently. Compared to the WT cells, the *4KR* mutant resected the DSB ends at a normal rate, while the *4KQ* mutant processed the ends faster in the distal region (Supplementary Figures S10A and B). This is likely attributed to reduced binding of the checkpoint adaptor protein Rad9, which is known to suppress DNA end resection (Supplementary Figure S10C) (70). Thus, the defect of HR repair in the *4KR* or *4KQ* mutant was not due to any defects in DNA end resection.

**Figure 4.**
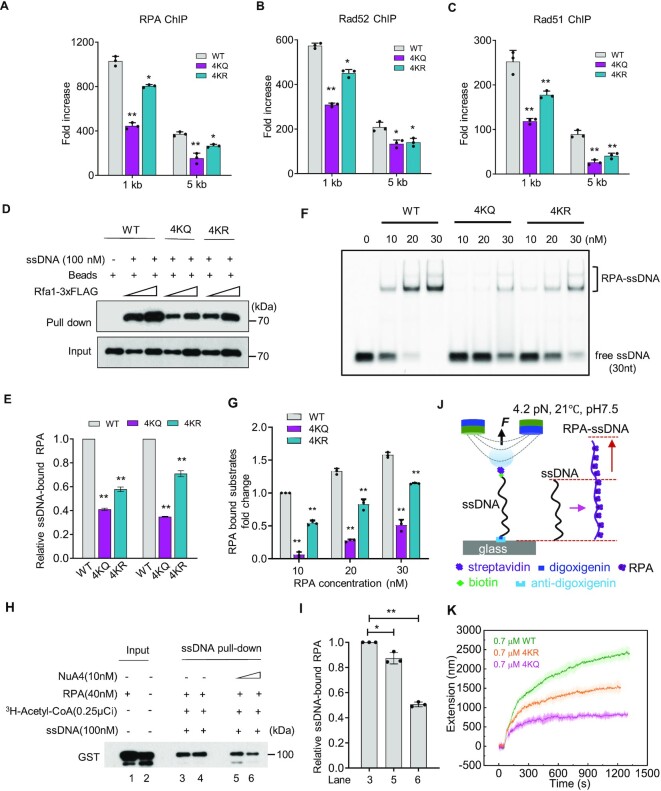
Proper RPA acetylation promotes RPA loading on ssDNA. (**A**–**C**) ChIP analysis of Rfa1-3xFLAG (**A**), Rad52-3xFLAG (**B**) and Rad51-3xFLAG (**C**) recruitment 4hr after DSB induction at indicated locations. (**D, E**) An ssDNA pull-down assay and quantification showing the effect of *4KQ* or *4KR* mutation on RPA binding to ssDNA. (**F, G**) An EMSA assay showing the binding of the WT or mutant RPA protein on ssDNA (30nt). Quantification of the relative RPA binding ability of indicated proteins is presented in (G). (**H, I**) An ssDNA pull-down assay and quantification showing the effect of RPA acetylation on its affinity to ssDNA. 100 nM of 5′-biotinylated ssDNA (90 nt) was immobilized to streptavidin beads for each reaction. (**J**) Scheme showing the single-molecule twister system for monitoring the kinetics of RPA assembly on ssDNA. (**K**) Single-molecule twister analysis indicating the effect of *4KQ* or *4KR* mutation on RPA assembly with ssDNA. Protein concentrations used are indicated. Error bars in this figure represent the standard deviation from at least three independent experiments. * *P*< 0.05, **P< 0.01 (Student's *t*-test).

Although ssDNA was efficiently produced, RPA loading at DSB ends was significantly impaired in the *4KQ* mutant and, to a lesser extent, in the *4KR* mutant (Figure 4A). RPA is required for efficient loading of Rad52 and Rad51 at DSB ends. As a result, the loading of Rad52 and Rad51 at DSBs was significantly impaired in the *4KQ* mutant and modestly decreased in the *4KR* mutant (Figures 4B and C). The more severe defect in Rad52 loading might explain the reduced SSA repair in the *4KQ* mutant (Figure 2E). However, both the recombinant WT and mutant Rfa1 proteins can efficiently interact with Rad51 or Rad52 *in vitro* (Supplementary Figures S11A and B), suggesting that the loading defects of Rad52 and Rad51 were caused by impaired RPA loading rather than any defects in the interaction between RPA and Rad52 or Rad51. Together, these results indicate that proper acetylation and deacetylation of RPA are required for proper loading of RPA, Rad52 and Rad51 at DNA breaks.

### Proper RPA acetylation regulates RPA binding to ssDNA

Next, we assessed the ssDNA-binding ability of the mutant RPA proteins. We incubated biotin-labeled ssDNA (30nt) coupled to magnetic beads with cell lysates from MMS-treated yeast cells to attract the endogenous RPA. Compared to the WT cells, both mutant proteins exhibited a reduced affinity to ssDNA, with the 4KQ mutant protein having a more severe defect (Figures 4D and E). It is worth noting that the 4KQ or 4KR mutation did not affect the RPA protein level or the assembly of the RPA complex, as the mutant Rfa1 can efficiently immunoprecipitate Rfa2 and Rfa3, as WT cells do (Supplementary Figures S11C and D). To confirm the result, we purified the WT and mutant RPA complexes from yeast cells and tested their ssDNA-binding abilities using the electrophoretic mobility shift assay (EMSA) (Supplementary Figure S12A). The addition of the WT RPA complex resulted in the shift of ssDNA in a dose-dependent manner, indicating the binding of RPA on ssDNA. In contrast, the 4KR mutant RPA complex exhibited reduced binding ability to ssDNA, and the defect was even more pronounced for the 4KQ mutant protein (Figures 4F and G). A similar result was obtained with a longer ssDNA (90 nt) substrate (Supplementary Figures S12B, C). Reduced formation of the RPA-ssDNA complex for the 4KQ and 4KR mutant RPA was not due to any degradation of ssDNA by potential contamination of nucleases since digestion of the RPA-ssDNA complex with protease K completely restored the levels of free ssDNA (Supplementary Figure S12D).

We then directly measured the impact of acetylation on the ssDNA-binding ability of RPA. Purified GST-tagged RPA complex was first incubated with the 5′-biotinylated ssDNA (90 nt), followed by the addition of the acetyltransferase NuA4 and acetyl-CoA to initiate the acetylation reaction. By streptavidin pull-down assay, we observed that the inclusion of NuA4 in the reaction impaired the binding of RPA on ssDNA in a dose-dependent manner (Figures 4H and I, lanes 3–6). This is not due to any potential degradation of ssDNA by the purified protein complex (Supplementary Figure S12E). Thus, these results suggest that direct acetylation of RPA reduces its ssDNA binding ability, as previously noted(54).

Finally, we employed single-molecule magnetic tweezers (MT) to monitor the ssDNA binding kinetics for the purified WT, 4KR, or 4KQ mutant Rfa1. We labeled 12.5 k nt ssDNA molecules with digoxigenin and biotin at the 5′- and 3′-ends, respectively, and stretched ssDNA molecules using MT at 4.2 pN, 21°C, and pH 7.5, as previously reported (Figure 4J) (34). We calculated the average values from multiple ssDNA molecules over time courses for each condition and plotted them as a time-extension curve. The addition of WT Rfa1 resulted in a striking extension of ssDNA, reflecting that RPA efficiently assembled with ssDNA. However, the assembly of the 4KR and, more severely, the 4KQ mutant protein on ssDNA was impaired (Figure 4K). Thus, we conclude that proper RPA acetylation and deacetylation are required for efficient RPA binding on ssDNA.

### Proper RPA acetylation cooperates with Rtt105 to regulate RPA nuclear import and HR repair

**Figure 5.**
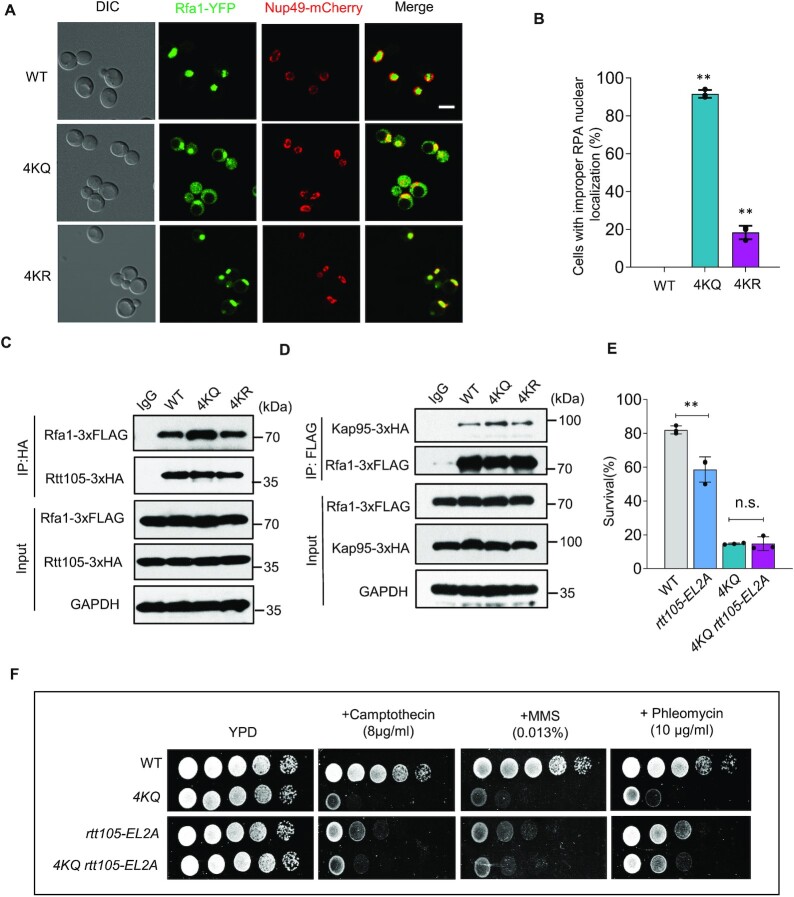
Constitutive RPA acetylation impacts RPA nuclear localization and its interaction with Rtt105. (**A, B**) Microscopy analysis and quantification of Rfa1-YFP nuclear localization in the WT, *4KQ*, or *4KR* cells. Nup49-mCherry was used as the marker of the nuclear membrane. (**C, D**) Immunoprecipitation showing the interaction between Rfa1-3xFLAG and Rtt105-3xHA or Kap95-3xHA in indicated cells. GAPDH was used as a control. (**E**) The survival rate for DSB repair by ectopic recombination in indicated cells. (**F**) DNA damage sensitivity test for indicated strains at indicated drug concentrations. Error bars in (B) and (E) represent the standard deviation from at least three independent experiments. n.s., no significance. **P< 0.01 (Student's *t*-test).

Next, we fused a yellow fluorescent protein (YFP) to the C-terminus of Rfa1 and examined whether proper acetylation affects RPA nuclear localization. As expected, the WT RPA is fully localized in the nucleus. Surprisingly, the 4KQ mutant protein localized in both cytoplasm and the nucleus (Figures 5A and B), as observed in cells lacking Rtt105, an RPA chaperone protein facilitating RPA nuclear import (32,34). Interestingly, ∼17% of *4KR* mutant cells also exhibited altered RPA subcellular localization (Figures 5A and B and Supplementary Figure S13). These results suggest that proper acetylation and deacetylation of RPA are required for its normal nuclear import.

Previous studies showed that Rtt105 cooperates with the importin Kap95 to regulate RPA nuclear import(32). Therefore, we tested whether the *4KR* or *4KQ* mutation affects RPA interaction with Rtt105 or Kap95. Interestingly, we found that the 4KQ mutant RPA exhibited an enhanced interaction with Rtt105 and, to a lesser extent, Kap95, as compared to the WT RPA (Figures 5C and D). In contrast, these interactions appeared to remain unaffected for the 4KR mutant protein. Consistently, using the microscale thermophoresis (MST) assay, we found that the 4KQ mutant RPA exhibited a higher affinity to 6xHis-Rtt105 (*K*_d_ ∼0.51 nM) than the WT RPA (*K*_d_ ∼1.06 nM) or the 4KR mutant RPA (*K*_d_ ∼1.17 nM) (Supplementary Figure S14). We speculated that the slightly enhanced association might prolong the occupancy of Rtt105 or Kap95 by the 4KQ mutant RPA, impairing the dynamic hand-on and hand-off of RPA upon importing into the nucleus, thus leading to improper RPA nuclear localization.

Rtt105 promotes high-fidelity DNA replication or repair via regulating RPA(34). We assessed the relationship between Rtt105 and RPA acetylation in regulating RPA functions. First, disruption of the Rtt105-RPA interaction by the *rtt105-E171A L172A* (*rtt105*-*EL2A*) mutation impaired DSB repair by ectopic recombination and the resistance to DNA damaging agents (Figures 5E and F) (32,34), while additional mutation of *rtt105*-*EL2A* in the *4KQ* mutant did not further reduce the HR repair rate and the drug resistance of the latter (Figures 5E and F), suggesting that Rtt105 and *4KQ* act epistatically in the DNA damage response or HR repair. Second, the *rtt105-EL2A* or *4KQ* single mutant and the double mutant exhibited similar phenotypes in mutation frequency and pattern (Figures 3A–F and Supplementary Figure S7), especially with the signature of micro-homology-mediated large deletions or duplications(34). Finally, both *4KQ* mutation and *RTT105* deletion can lead to improper RPA nuclear localization. These results suggest that proper RPA acetylation cooperates with Rtt105 to facilitate RPA nuclear localization, HR repair, and the response to DNA damage.

### Proper acetylation and deacetylation of human RPA promotes RPA and RAD51 loading at DNA breaks

**Figure 6.**
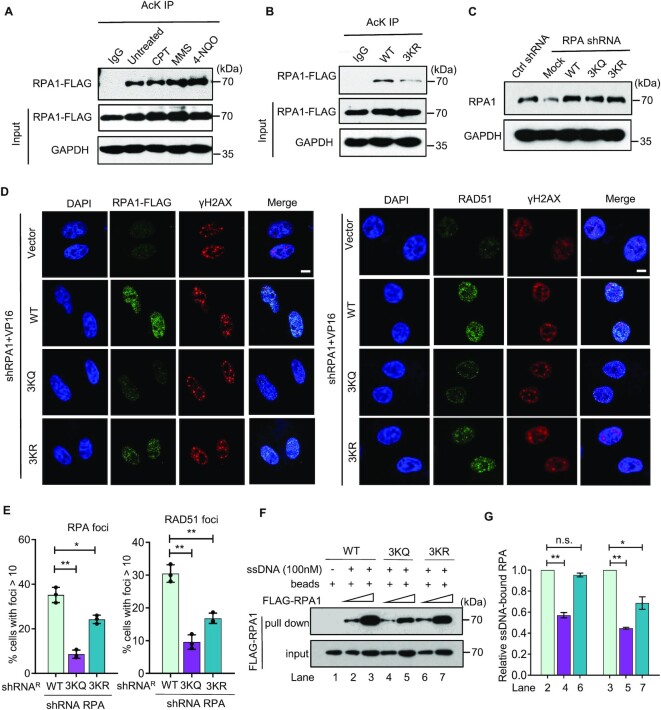
Proper acetylation and deacetylation of human RPA promote RPA and RAD51 loading. (**A**) Immunoprecipitation and western blot showing acetylation of the ectopically expressed RPA1 in response to the DNA damage in HEK293T cells. An anti-acetyl-lysine antibody was used to carry out immunoprecipitations, followed by probing with an anti-FLAG antibody. (**B**) Immunoprecipitation showing acetylation of the ectopically expressed WT or 3KR-RPA1 in HEK293T cells. (**C**) Western blot indicating RPA1 levels in HEK293T cells transfected with the control shRNA or in cells with a vector or reconstituted WT, 3KQ or 3KR RPA1. GAPDH was used as a loading control. (**D, E**) Immunostaining and quantification of RPA1 or RAD51 foci number post VP16 treatment (5 μM, 1 h) in HeLa cells. γH2AX was stained as a marker of the DNA damage, and the relative RPA and RAD51 foci number was normalized to the corresponding γH2AX foci number. Scale bar: 10 μm. (**F, G**) An ssDNA pull-down assay and the quantification showing the effect of *3KQ* or *3KR* mutation on the ssDNA-binding affinity of human RPA. Error bars indicate the standard deviation from three independent experiments. Statistical analysis was calculated with the Student's *t*-test. n.s., no significance. * *P*< 0.05, ** *P*< 0.01.

Notably, three of the four acetylated lysines in yeast Rfa1 are conserved across species, which are equivalent to K259, K458 and K489 in human RPA1, the largest subunit of the human RPA complex (Supplementary Figure 1F). Except for the UV-induced acetylation on K163 (55,56), several other residues, including K259, K458 and K489 of human RPA1, were also identified as acetylated sites by mass spectrometry studies (54,83–85). To confirm this result, we immunoprecipitated acetylated proteins with an anti-pan-acetyl-lysine antibody from HEK293T cells expressing the FLAG-tagged RPA1 and examined RPA acetylation using an anti-FLAG antibody. In line with previous studies, we successfully detected the acetylation of RPA1 in the presence of trichostatin A (TSA) and nicotinamide (NAM), the inhibitors of deacetylases. In addition, we noted that the signal was increased upon treatment with the DNA-damaging agents MMS or 4NQO but not CPT (Figure 6A) (55,56). Notably, we found that RPA acetylation was significantly reduced in cells expressing the plasmid-borne *RPA1-3KR* (K259R, K458R and K489R) mutant allele, suggesting that these residues are indeed acetylated in human cells (Figure 6B).

Guided by our findings in yeast, we evaluated whether RPA acetylation affects its loading and HR repair in human cells. First, we depleted endogenous RPA1 in Hela cells using a small hairpin interfering RNA (shRNA) and transfected these cells with either an empty vector or a plasmid expressing the shRNA-resistant WT, *3KR*- or *3KQ*-*RPA1* allele (Figure 6C). We observed that RPA foci formation was impaired in the reconstituted *3KR* cells upon VP16 (etoposide) treatment that can induce DSBs, and the defect was more pronounced in the reconstituted *3KQ* cells as compared to the corresponding WT cells (Figures 6D and E). However, unlike the yeast *4KQ* mutant, the human *3KR* or *3KQ* mutation did not change RPA nuclear localization (Figure 6D). To confirm the above result, we incubated biotin-labeled ssDNA (30nt) coupled to magnetic beads with the whole cell extracts derived from VP16-treated HEK293T cells expressing the FLAG-tagged WT or mutant *RPA1* allele and performed an ssDNA pull-down assay. Notably, compared to the WT RPA, the 3KQ mutant protein exhibited an evident defect in binding ssDNA at both lower and higher concentrations, while the 3KR mutant protein only displayed a defect in binding ssDNA at the higher concentration (Figures 6F and G). Consequently, RAD51 foci formation was impaired in both *3KR* and *3KQ* reconstituted cells upon VP16 treatment (Figures 6D and E).

### Proper acetylation and deacetylation of human RPA promote efficient HR repair

**Figure 7.**
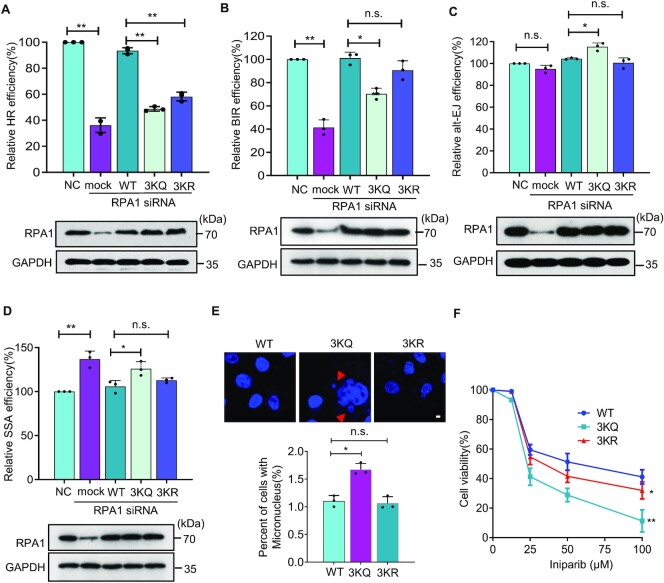
Proper acetylation and deacetylation of human RPA promote accurate DSB repair by HR while suppressing the SSA or alt-EJ pathway. (**A–D**) Plot showing the relative efficiency of HR, BIR, alt-EJ, and SSA, respectively, measured with the corresponding GFP reporters for indicated cell lines. The corresponding RPA1 protein level was detected by Western blot and shown at the bottom of each plot. GAPDH served as a loading control. (**E**) Representative images showing micronuclei in indicated Hela cells. Arrows indicate micronuclei stained by DAPI. The level of micronuclei in the reconstituted WT, *3KQ* or *3KR* cells was quantified. Quantifications of A–E are the average values of three independent experiments. Data were analyzed by Students’ *t-*test. n.s., no significance. * *P*< 0.05, ** *P*< 0.01. Scale bar: 10 μm. (**F**) Survival curves for RPA1 siRNA cells complemented with the plasmid expressing the WT, *3KQ* or *3KR* plasmid upon exposure to iniparib treatment. Data were analyzed by two-way ANOVA, *n* = 3. *P< 0.05, ** *P*< 0.01.

We then assessed the effect of RPA acetylation on HR repair using the I-*Sce*I DR-GFP reporter in U2OS cells (62). In this reporter, repair of the I-*Sce*I-induced DSB by HR restores the fluorescent signal of EGFP that can be monitored by flow cytometry (Supplementary Figure S15A). First, we depleted the endogenous RPA1 with siRNA and complemented the cells with an empty vector or a plasmid; expressing a siRNA-resistant WT, *3KQ*, or *3KR* RPA1 allele. The reconstituted WT cells were fully proficient in repairing the I-*Sce*I-induced DSB by HR, while the reconstituted *3KQ* or *3KR* mutant allele exhibited a significant defect in the HR repair (Figure 7A). Next, we evaluated the effect of *3KQ* or *3KR* mutation on BIR using the EGFP-BIR-5085 reporter system in U2OS cells(64). In this reporter, the I-*Sce*I-induced DSB on the recipient chromatid can be repaired by BIR by copying the sequence on the template chromatid to the end via synthesis-dependent strand annealing (SDSA) or through copying a short segment of the donor template followed by end joining with the other end of the DSB (Supplementary Figure S15B). Compared to the reconstituted WT cells, the reconstituted *3KQ* but not *3KR* cells exhibited an attenuated BIR repair efficiency (Figure 7B). Thus, proper RPA acetylation and deacetylation are required for efficient RPA and RAD51 loading and DSB repair by gene conversion or BIR.

### Constitutive acetylation of human RPA increases alt-EJ, SSA and genome instability

In parallel, we measured the effect of *3KQ* or *3KR* mutation on DSB repair by alt-EJ in U2OS cells that carry an EGFP-alt-EJ reporter and stably express doxycycline (DOX)-inducible Cas9 and sgRNA (63). sgRNA directs the Cas9 endonuclease to the I-*Sce*I recognition site to generate a DSB upon induction with DOX (Supplementary Figure S15C). We noted that the reconstituted *3KQ* but not the *3KR* cells had a slight increase in alt-EJ efficiency compared to the corresponding WT cells (Figure 7C). Similarly, using an established SSA-EGFP reporter in U2OS cells (65), we measured the SSA-mediated repair of the I-*Sce*I-induced DSB (Supplementary Figure S15D). We found that the reconstituted *3KR* cells did not affect SSA efficiency, while the reconstituted *3KQ* cells exhibited a significant increase in SSA repair (Figure 7D). Accordingly, the Hela cells expressing the *3KQ* mutant allele had an increased level of micronuclei, an indicator of chromosome loss (Figure 7E).

In line with the role of proper RPA acetylation in DSB repair, cells expressing the *3KQ* mutant allele had a severe defect in resistance to Iniparib, an anti-cancer agent, and the *3KR* mutant lines also exhibited a reduced resistance to the drug, albeit to a lesser extent (Figure 7F). Together, these results suggest that proper acetylation and deacetylation of RPA are required to promote DSB repair by the accurate gene conversion or BIR while suppressing the repair by the deleterious alt-EJ or SSA mechanism in human cells. Otherwise, it leads to genome instability. Thus, RPA acetylation appears to be a conserved regulatory mechanism in guarding genome stability.

Since replication gaps and the RPA level are important determinants impacting the killing efficiency of BRCA deficiency cells by PARP inhibitors(86), we examined the response of the reconstituted WT, *3KQ*, and *3KR* Hela cells to replication stresses. Both the WT and the 3KR or 3KQ mutant RPA can localize to ssDNA gaps at stalled forks induced by hydroxyurea (HU, 5 mM) at a comparable level, and all these cell lines can respond and recover from the HU treatment at similar kinetics, as reflected by the formation and removal of γH2AX foci (Supplementary Figures S16A–D). Thus, proper acetylation and deacetylation of RPA appear particularly important for DNA repair rather than replication stresses in the BRCA proficient cells.

## DISCUSSION

How RPA is finely regulated to ensure accurate DNA replication and repair remains poorly understood. In this study, we found that proper acetylation and deacetylation of RPA suppresses micro-homology-mediated large deletions or duplications and facilitates the accurate DSB repair by gene conversion or BIR while discriminating the error-prone SSA or alt-EJ pathway in yeast (Supplementary Figures S17A, B). In parallel, we showed that proper RPA acetylation or deacetylation also facilitates RPA and RAD51 loading, gene conversion, and BIR while inhibiting alt-EJ and SSA in human cells. Mechanistically, we showed that proper acetylation regulates the nuclear import or ssDNA-binding ability of RPA. Thus, we reveal a conserved function of RPA acetylation in promoting high-fidelity DNA replication or repair and regulating DSB repair pathway choice.

We found that RPA acetylation acts via at least two layers of mechanisms in yeast. First, proper RPA acetylation is required for normal RPA nuclear localization. RPA is primarily localized in the nucleus. However, how and why acetylation affects RPA unclear localization is unclear. One possibility is that RPA accompanies a small fraction of damaged DNA that is disassociated from chromatin and has been passively exported to the cytoplasm. Alternatively, RPA acetylation may affect its turnover, as noted for the DNA damage-induced Sae2 acetylation that promotes Sae2 degradation via autophagy (87). Acetylation-dependent control of protein subcellular localization has also been observed for other proteins, such as human MDM2 and E1A (88,89). However, mutation of the equivalent sites in human RPA1 does not affect RPA nuclear localization, suggesting that RPA nuclear transport is differentially regulated between yeast and human. Second, we showed that proper acetylation and deacetylation of RPA facilitate its binding to ssDNA. These acetylation sites appear on the surfaces of the RPA structure and are close to the ssDNA binding surfaces (Supplementary Figure S1G), making it possible to regulate RPA ssDNA affinity by acetylation. Acetylation of human Dna2 and FEN1 also regulates their DNA binding abilities (90,91). Notably, the *4KQ* mutant is more defective than the *4KR* mutant in all tested phenotypes, suggesting that timely deacetylation of RPA is more critical. This is consistent with the extent of their defects in ssDNA binding or nuclear localization. However, it should be noted that arginine and glutamine may not always accurately mimic lysine and acetyl-lysine, respectively. Therefore, we cannot exclude the possibility that the defects in the extreme *4KQ*/*3KQ* or *4KR*/*3KR* mutants are partially caused by the mutation itself. The human *3KR* mutation causes a moderate defect in RPA loading and HR repair, while it does not impact BIR, alt-EJ, or SSA. BIR repairs one-ended DSBs, while alt-EJ and SSA join or anneal short complementary sequences, respectively. These repair mechanisms occur at a much lower frequency than typical HR repair and usually involve a lesser amount of ssDNA. This probably explains why the 3KR mutation, which has a moderate defect in RPA loading, does not affect the repair by BIR, alt-EJ or SSA.

Spontaneous micro-homology-mediated deletions or duplications are believed to associate with replication slippage or the repair by SSA. These types of mutations were seen in cells with mutations in DNA replication genes, such as *RFA1*, *RAD27*, *POL3*, *POL32* and *RTT105* (34,74–79). A potential mechanism was proposed by Tishkoff et al. to explain how this type of event is generated (77). They proposed that the lagging strand DNA polymerase Pol δ extends DNA into the downstream Okazaki fragment and displaces it during DNA synthesis, generating a 5′-flap structure that can lead to a short duplication of the DNA sequence if left unprocessed. The 5′-ssDNA overhang can potentially be filled in by DNA synthesis and subsequently resected to expose the short repeats at the 3′-end for SSA (77). The pairing of the repeats out of the register can cause either duplications or deletions (77). The nucleases Dna2 and Rad27 cooperate to process the 5′-flaps at Okazaki fragments, while RPA is known to regulate the two enzymes(14). Therefore, in the *4KR* or *4KQ* mutant, reduced RPA binding on ssDNA may lead to aberrant processing of the displaced flaps or misalignment of short homologies, causing duplications or deletions. Micro-homology-mediated large duplications or deletions often occur in human cancers (2–5). Our results and previous work suggest that mutations in RPA or its regulator could contribute to such genome rearrangements (34,74).

RPA plays a variety of functions at multiple steps of replication, repair, and recombination. Therefore, the binding of RPA with ssDNA or proteins must be highly dynamic to allow efficient binding yet timely disassociation. However, how these interactions are dynamically regulated in the chromatin context is poorly understood. Here, we found that constitutive RPA acetylation impaired its ssDNA binding ability yet enhanced its affinity with Rtt105. Thus, proper RPA acetylation represents an important mechanism for regulating RPA activities or interactions with its partners. This regulation might facilitate passing ssDNA substrates to downstream replication or repair proteins or channeling ssDNA intermediates to a correct repair pathway.

The ssDNA–RPA complex is an important biological intermediate formed throughout the life of cells(9,92). Due to its broad functions in DNA metabolism, RPA is tightly associated with carcinogenesis (9,20–23). RPA depletion or exhaustion can lead to genome instability, compromised DNA repair, and reduced resistance to radiation or chemotherapeutic agents in cancer cells (9,93). Therefore, both the nuclear import and proper binding of RPA on ssDNA are essential for avoiding mutations or cancer. Notably, both aspects of RPA are affected by its acetylation, underscoring the importance of this regulation in guarding genome integrity and preventing cancer.

## DATA AVAILABILITY

The data underlying this article are available in the article and in the online supplementary data.

## Supplementary Material

gkad291_Supplemental_File
