# Supplementary material for: Proper RPA acetylation promotes accurate DNA replication and repair
Source: Nucleic Acids Res. 2023 May 4;51(11):5565–83. doi: 10.1093/nar/gkad291 (PMC10287905; doi:10.1093/nar/gkad291)
Supplement: gkad291_Supplemental_File [file gkad291_supplemental_file.pdf]

Figure S1

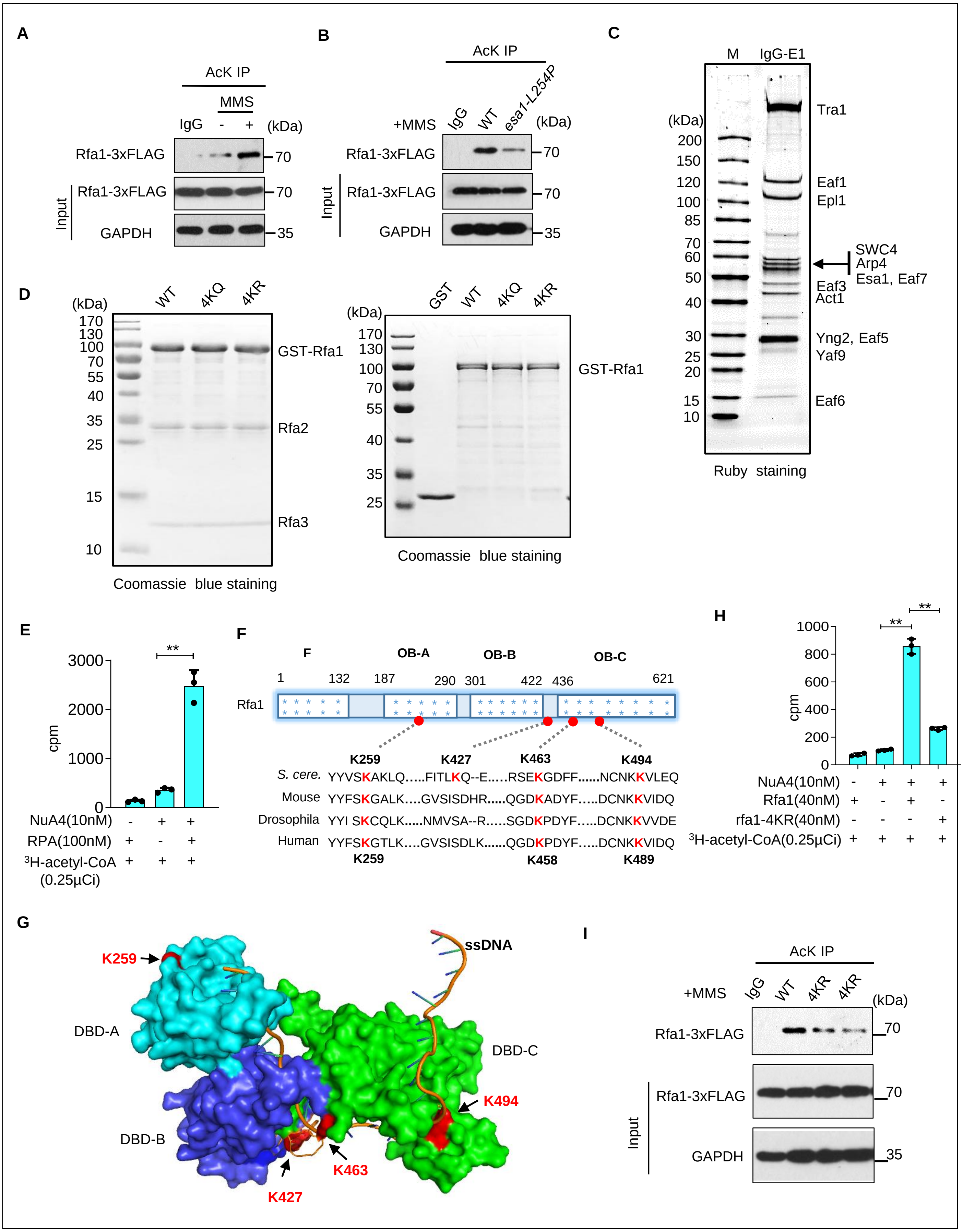
A
AcK IP
MMS
IgG - + (kDa)
Rfa1-3xFLAG 70
Input
Rfa1-3xFLAG 70
GAPDH 35
B
AcK IP
+MMS IgG WT esa1-L254P (kDa)
Rfa1-3xFLAG 70
Input
Rfa1-3xFLAG 70
GAPDH 35
C
M IgG-E1
(kDa)
200 150 120 100 85 70 60 50 40 30 25 20 15 10
Tra1
Eaf1
Epl1
SWC4
Arp4
Esa1, Eaf7
Eaf3
Act1
Yng2, Eaf5
Yaf9
Eaf6
Ruby staining
D
(kDa) WT 4KQ 4KR
170 130 100 70 55 40 35 25 15 10
GST-Rfa1
Rfa2
Rfa3
Coomassie blue staining
(kDa) GST WT 4KQ 4KR
170 130 100 70 55 40 35 25
GST-Rfa1
Coomassie blue staining
E
cpm
3000 2000 1000 0
**
NuA4(10nM) - + +
RPA(100nM) + - +
³H-acetyl-CoA (0.25µCi) + + +
F
F OB-A OB-B OB-C
1 132 187 290 301 422 436 621
Rfa1
K259 K427 K463 K494
S. cere. YYVSKAKLQ.....FITLKQ--E.....RSEKGDFF......NCNKKVLEQ
Mouse YYFSKGALK....GVSISDHR......QGDKADYF.....DCNKKVIDQ
Drosophila YYI SKCQLK.....NMVSA--R.....SGDKPDYF.....DCNKKVVDE
Human YYFSKGTLK....GVSISDLK.......QGDKPDYF.....DCNKKVIDQ
K259 K458 K489
H
cpm
1000 800 600 400 200 0
** **
NuA4(10nM) - + + +
Rfa1(40nM) + - + -
rfa1-4KR(40nM) - - - +
³H-acetyl-CoA(0.25µCi) + + + +
G
K259
ssDNA
DBD-A
DBD-C
DBD-B
K494
K463
K427
I
AcK IP
+MMS IgG WT 4KR 4KR (kDa)
Rfa1-3xFLAG 70
Input
Rfa1-3xFLAG 70
GAPDH 35

Supplementary Figure S1. RPA is acetylated by NuA4 in vivo and in vitro. (**A**) Immunoprecipitation and Western blot analysis showing Rfa1 acetylation in the absence or presence of 0.1% MMS treatment (1hr). The anti-acetyl-lysine antibody was used to carry out immunoprecipitations, followed by immunoblotting with the anti-FLAG antibody. (**B**) Immunoprecipitation and Western blot analysis showing Rfa1 acetylation in the WT or *esa1-L254P* mutant cells upon MMS treatment. (**C**) SDS-PAGE and Ruby staining of the NuA4 complex purified from yeast cells. (**D**) SDS-PAGE and Coomassie blue staining of the GST-RPA complex or GST-Rfa1 purified from E.coli. (**E,H**) In vitro acetylation for the RPA complex (E) or the WT or 4KR mutant Rfa1(H), as measured by liquid scintillator counter. (**F**) Scheme showing the conservation of the acetylated lysines in Rfa1 from different species. Red dots indicate the locations of acetylated lysines in yeast Rfa1. (**G**) A model showing the structure of yeast Rfa1 and the locations of the acetylated lysines. The acetylation sites are marked in red and indicated by arrows. (**I**) Immunoprecipitation analysis of Rfa1 acetylation in the WT or *4KR* mutant cells upon MMS treatment. Error bars in (E) and (H) represent the standard deviation from three independent experiments. Statistical analysis was calculated with the Student *t*-test. ** $p < 0.01$.

Figure S2

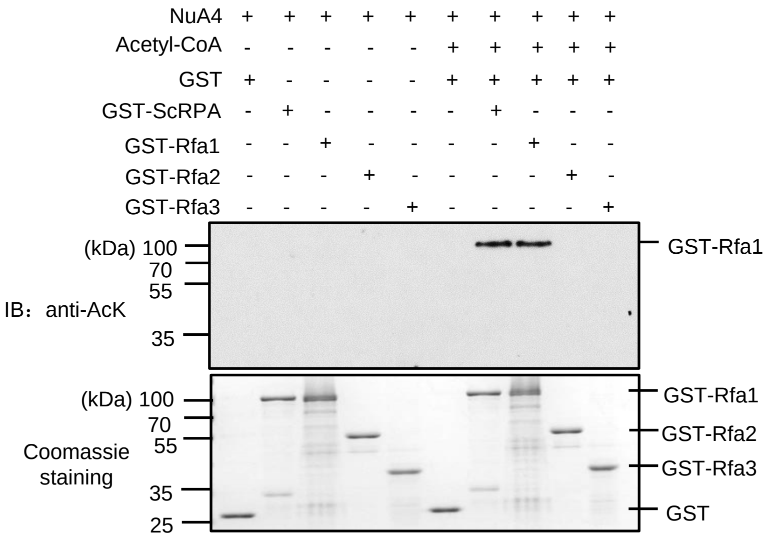

Supplementary Figure S2. Rfa1 is the primary subunit acetylated by NuA4 in the RPA complex. An in vitro acetylation assay testing the acetylation of Rfa1, Rfa2 or Rfa3 by NuA4. The products were detected by Western blot using the anti-acetyl-lysine antibody. The bottom panel shows the Coomassie blue staining of the purified GST-RPA complex or GST-tagged Rfa1, Rfa2 or Rfa3 subunit used for the experiment.

Figure S3

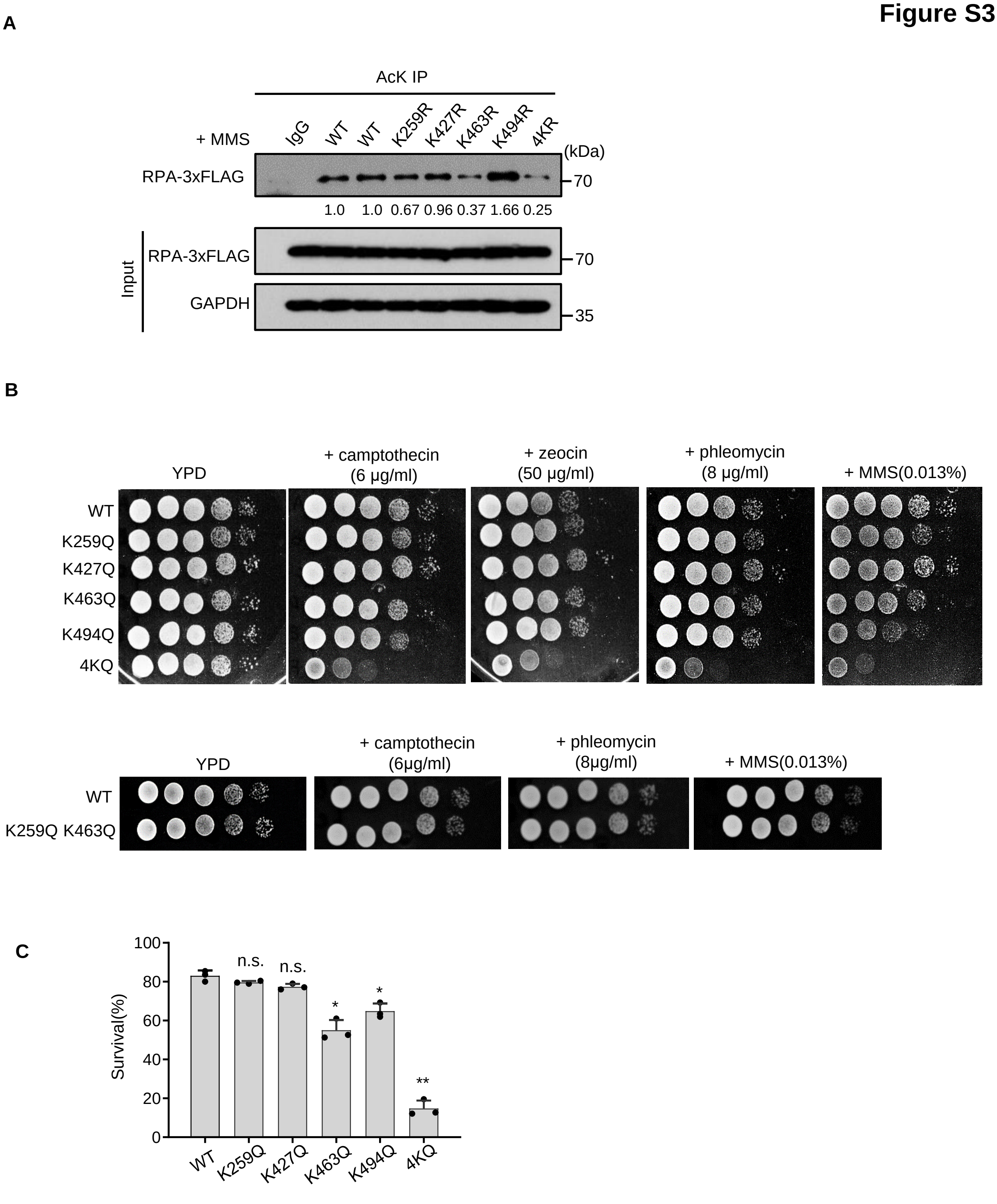

Supplementary Figure S3. Acetylation on multiple sites of Rfa1 functions collectively to promote DNA damage response and HR repair. A. Immunoprecipitation and Western blot analysis showing RPA acetylation in indicated yeast cells. Immunoprecipitation was performed with the anti-acetyl-lysine antibody. The products were detected by Western blot using an anti-FLAG antibody. The relative level of RPA acetylation is indicated for each strain. **B.** DNA damage sensitivity test for indicated strains at indicated drug concentrations. Cells were grown on YPD (1% yeast extract, 2% peptone, and 2% dextrose) plates. **C**. Survival rate of DSB repair by ectopic recombination in indicated cells. Error bars denote standard deviations from three independent experiments. Statistical analysis was calculated with the Student *t*-test. * $p < 0.05$, ** $p < 0.01$.

Figure S4

A

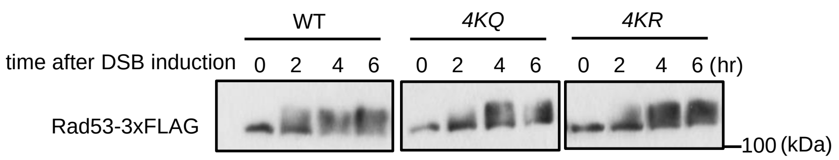

B

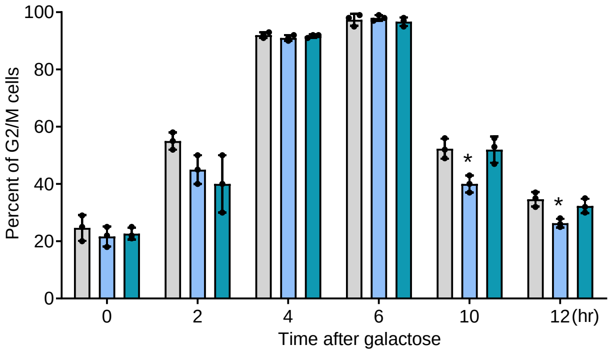

Supplementary Figure S4. The impact of 4KQ or 4KR mutation on DNA damage checkpoint activation. A. Western blot showing Rad53 phosphorylation, an indicator of checkpoint activation, following DSB induction. The HO cut was induced by the addition of galactose. **B.** Graph showing the percent of G2/M cells following DSB induction. Samples were collected at indicated time points after galactose induction. Error bars denote standard deviations from three independent experiments. Statistical analysis was calculated with the Student *t*-test. * $p < 0.05$.

Figure S5

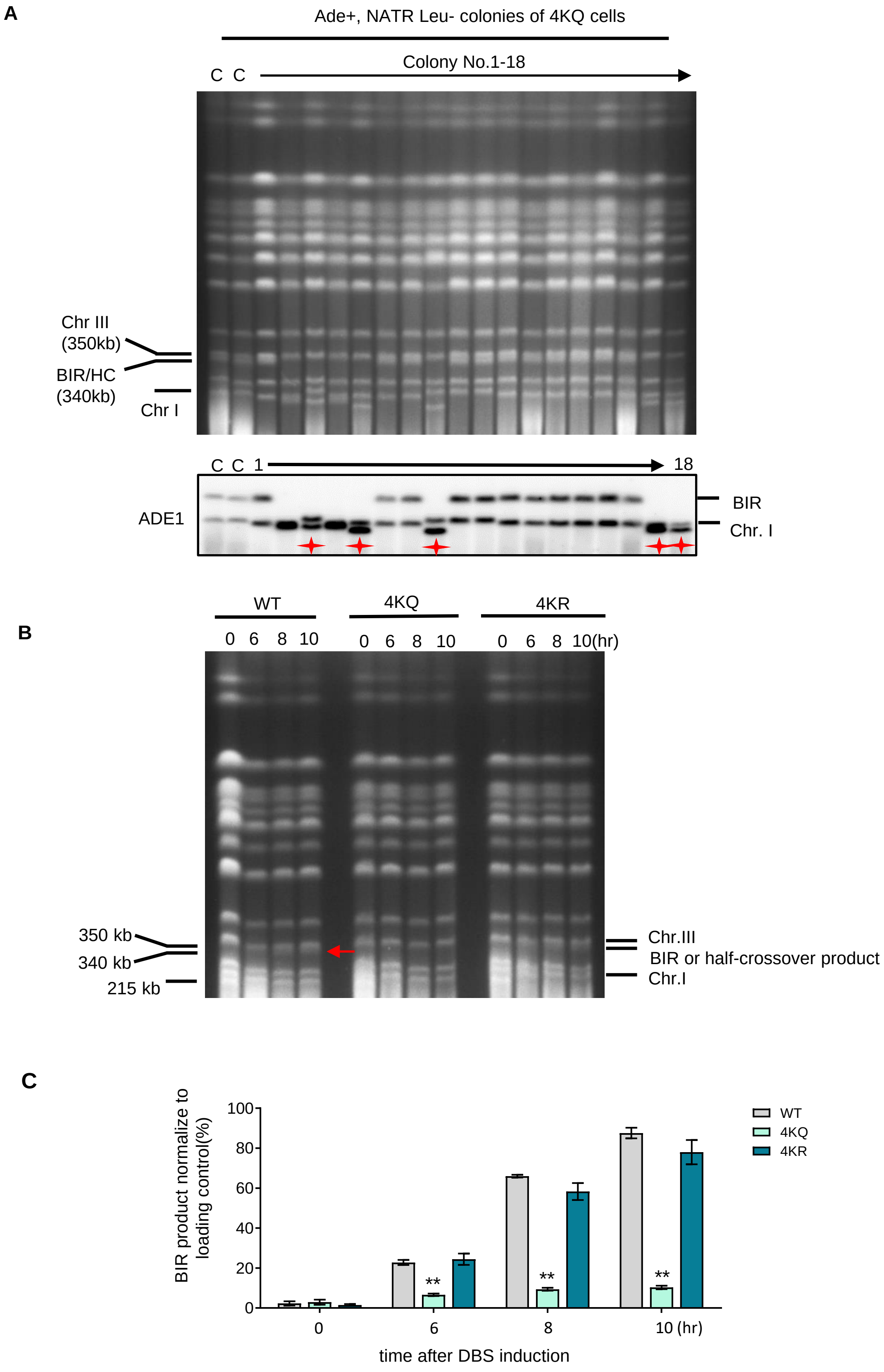

Supplementary Figure S5. Analysis of BIR products and repair kinetics. A. PFGE and Southern blot analysis of recombination products from Ade$^+$, NAT^R Leu$^-$ colonies for the *4KQ* mutant cells. Recombination events associated with the rearrangement of the recipient chromosome are indicated. **B**. BIR kinetics revealed by PFGE for the WT, *4KQ,* and *4KR* cells. The BIR product is indicated by an arrow**. C**. Quantification of BIR kinetics in Figure 1I. Error bars denote standard deviations from three independent experiments. Statistical analysis was calculated with the Student *t*-test. ** $p < 0.01$.

Figure S6

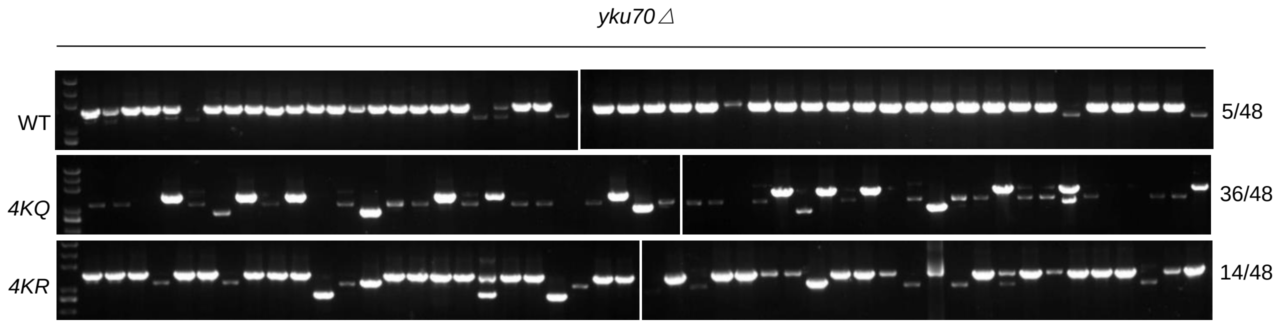

Supplementary Figure S6. Analysis of the alt-EJ repair products by PCR for indicated strains. A pair of PCR primers spanning the HO cut site was used to amplify the repair products. PCR products were separated on a 1% agarose gel. The number of deletion events detected is indicated.

Figure S7

Mutation spectrum for CanR colonies derived from WT cells

```
1      ATGACAAATTCAAAAGAAGACGCCGACATAGAGGAGAAGCATATGTACAATGAGCCGGTCACAACCCTCTTTCACGACGTTGAAGCTTCA
1      M T N S K E D A D I E E K H M Y N E P V T T L F H D V E A S

       A>T C>A T>G G>A T>A G>A -G G>A
91     CAAACACACCACAGACGTGGGTCAATACCATTAAAAGATGAGAAAAGTAAAGAATTGTATCCATTGCGCTCTTTCCCGACGAGAGTAAAT
31     Q T H H R R G S I P L K D E K S K E L Y P L R S F P T R V N

       C>G G>A G>T G>A G>A A>G G>A
181    GGCGAGGATACGTTCTCTATGGAGGATGGCATAGGTGATGAAGATGAAGGAGAAGTACAGAACGCTGAAGTGAAGAGAGAGCTTAAGCAA
61     G E D T F S M E D G I G D E D E G E V Q N A E V K R E L K Q

       G>T T>C G>A G>T -G C>A A>T G>C
271    AGACATATTGGTATGATTGCCCTTGGTGGTACTATTGGTACAGGTCTTTTCATTGGTTTATCCACACCTCTGACCAACGCCGGCCCAGTG
91     R H I G M I A L G G T I G T G L F I G L S T P L T N A G P V

       C>A T>G G>A G>A -T C>A
361    GGCGCTCTTATATCATATTTATTTATGGGTTCTTTGGCATATTCTGTCACGCAGTCCTTGGGTGAAATGGCTACATTCATCCCTGTTACA
121    G A L I S Y L F M G S L A Y S V T Q S L G E M A T F I P V T

       C>T G>T
451    TCCTCTTTCACAGTGTTCTCACAAAGATTCCTTTCTCCAGCATTTGGTGCGGCCAATGGTTACATGTATTGGTTTTCTTGGGCAATCACT
151    S S F T V F S Q R F L S P A F G A A N G Y M Y W F S W A I T

541    TTTGCCCTGGAACTTAGTGTAGTTGGCCAAGTCATTCAATTTTGGACGTACAAAGTTCCACTGGCGGCATGGATTAGTATTTTTTGGGTA
181    F A L E L S V V G Q V I Q F W T Y K V P L A A W I S I F W V

       +C -A C>A G>A T>G G>T(2) G>A A>C G>T G>A
631    ATTATCACCAATAATGAACTTGTTCCCTGTCAAATATTACGGTGAATTCGAGTTCTGGGTCGCTTCCATCAAAGTTTTAGCCATTATCGGG
211    I I T I M N L F P V K Y Y G E F E F W V A S I K V L A I I G

       -T -G G>T(2) T>C -G G>A -G
721    TTTCTAATATACTGTTTTTGTATGGTTTGTGGTGCTGGGGTTACCGGCCCAGTTGGATTCCGTTATTGGAGAAACCCAGGTGCCTGGGGT
241    F L I Y C F C M V C G A G V T G P V G F R Y W R N P G A W G

       -A(2) A>C G>A -A -G A>C T>A G>C -T G>T G>A C>T C>A
811    CCAGGTATAATATCTAAGGATAAAAACGAAGGGGAGGTTCTTAGGTTGGGTTTCCTCTTTGATTAACGCTGCCTTCACATTTCAAGGTACT
271    P G I I S K D K N E G R F L G W V S S L I N A A F T F Q G T

       T>G C>T(2) G>A
901    GAACTAGTTGGTATCACTGCTGGTGAAGCTGCAAACCCCAGAAAATCCGTTCCAAGAGCCATCAAAAAAGTTGTTTTCCGTATCTTAACC
301    E L V G I T A G E A A N P R K S V P R A I +A K K V V F R I L T

       G>A -T +G C>A C>A(2) -C C>A
991    TTCTACATTGGCTCTCTATTATTCATTGGACTTTTAGTTCCATACAATGACCCTAAACTAACACAATCTACTTCCTACGTTTCTACTTCT
331    F Y I G S L L F I G L L V P Y N D P K L T Q S T S Y V S T S

       G>T -T -T(2)
1081   CCCTTTATTATTGCTATTGAGAACTCTGGTACAAAGGTTTTGCCACATATCTTCAACGCTGTTATCTTAACAACCATTATTTCTGCCGCA
361    P F I I A I E N S G T K V L P H I F N A V I L T T I I S A A

       G>C G>C T>C
1171   AATTCAAATATTTACGTTGGTTCCCGTATTTTATTTGGTCTATCAAAGAACAAGTTGGCTCCTAAATTCCTGTCAAGGACCACCAAAGGT
391    N S N I Y V G S R I L F G L S K N K L A P K F L S R T T K G

       G>T C>A G>T G>T
1261   GGTGTTCCATACATTGCAGTTTTCGTTACTGCTGCATTTGGCGCTTTGGCTTACATGGAGACATCTACTGGTGGTGACAAAGTTTTCGAA
421    G V P Y I A V F V T A A F G A L A Y M E T S T G G D K V F E

       G>T -C +T G>A C>T
1351   TGGCTATTAAATATCACTGGTGTTGCAGGCTTTTTTGCATGGTTATTTATCTCAATCTCGCACATCAGATTTATGCAAGCTTTGAAATAC
451    W L L N I T G V A G F F A W L F I S I S H I R F M Q A L K Y

       C>A T>A
1441   CGTGGCATCTCTCGTGACGAGTTACCATTTAAAGCTAAATTAATGCCCGGCTTGGCTTATTATGCGGCCACATTTATGACGATCATTATC
481    R G I S R D E L P F K A K L M P G L A Y Y A A T F M T I I I

1531   ATTATTCAAGGTTTCACGGCTTTTGCACCAAAATTCAATGGTGTTAGCTTTGCTGCCGCCTATATCTCTGTTTTCCTGTTCTTAGCTGTT
511    I I Q G F T A F A P K F N G V S F A A A Y I S V F L F L A V

       -T
1621   TGGATCTTATTTCAATGCATATTCAGATGCAGATTTATTTGGAAGATTGGAGATGTCGACATCGATTCCGATAGAAGAGACATTGAGGCA
541    W I L F Q C I F R C R F I W K I G D V D I D S D R R D I E A

1711   ATTGTATGGGAAGATCATGAACCAAAGACTTTTTGGGACAAATTTTGGAATGTTGTAGCATAG
571    I V W E D H E P K T F W D K F W N V V A *
```

Figure S7 continued

Mutation spectrum for CanR colonies derived from 4KQ cells

1 ATGACAAATTCAAAAGAAGACGCCGACATAGAGGAGAAGCATATGTACAATGAGCCGGTCACAACCCTCTTTCACGACGTTGAAGCTTCA
1 M T N S K E D A D I E E K H M Y N E P V T T L F H D V E A S

C>G G>T
91 CAAACACACCACAGACGTGGGTCAATACCATTAAAAGATGAGAAAAGTAAAGAATTGTATCCATTGCGCTCTTTCCCGACGAGAGTAAAT
31 Q T H H R R G S I P L K D E K S K E L Y P L R S F P T R V N

A>G -AG
181 GGCGAGGATACGTTCTCTATGGAGGATGGCATAGGTGATGAAGATGAAGGAGAAGTACAGAACGCTGAAGTGAAGAGAGAGCTTAAGCAA
61 G E D T F S M E D G I G D E D E G E V Q N A E V K R E L K Q

271 AGACATATTGGTATGATTGCCCTTGGTGGTACTATTGGTACAGGTCTTTTCATTGGTTTATCCACACCTCTGACCAACGCCGGCCCAGTG
91 R H I G M I A L G G T I G T G L F I G L S T P L T N A G P V

395-529 (-135bp) 396-458 (-63bp) -G C>T
361 GGCGCTCTTATATCATATTTATTTATGGGTTCTTTGGCATATTCTGTCACGCAGTCCTTGGGTGAAATGGCTACATTCATCCCTGTTACA
121 G A L I S Y L F M G S L A Y S V T Q S L G E M A T F I P V T

533-628 (-96bp)(2) G>A G>C -C A>C C>A
451 TCCTCTTTCACAGTGTTCTCACAAAGATTCCTTTCTCCAGCATTTGGTGCGGCCAATGGTTACATGTATTGGTTTTCTTGGGCAATCACT
151 S S F T V F S Q R F L S P A F G A A N G Y M Y W F S W A I T

C>T -T G>T -A G>T
541 TTTGCCCTGGAACTTAGTGTAGTTGGCCAAGTCATTCAATTTTGGACGTACAAAGTTCCACTGGCGGCATGGATTAGTATTTTTTGGGTA
181 F A L E L S V V G Q V I Q F W T Y K V P L A A W I S I F W V

G>C G>C G>T G>A -T
631 ATTATCACAATAATGAACTTGTTCCCTGTCAAATATTACGGTGAATTCGAGTTCTGGGTCGCTTCCATCAAAGTTTTAGCCATTATCGGG
211 I I T I M N L F P V K Y Y G E F E F W V A S I K V L A I I G

-G +CTT G>C -G -G
721 TTTCTAATATACTGTTTTTGTATGGTTTGTGGTGCTGGGGTTACCGGCCCAGTTGGATTCCGTTATTGGAGAAACCCAGGTGCCTGGGGT
241 F L I Y C F C M V C G A G V T G P V G F R Y W R N P G A W G

-G(2) G>A G>C(3) -C C>A(2) G>T G>A
811 CCAGGTATAATATCTAAGGATAAAAACGAAGGGAGGTTCTTAGGTTGGGTTTCCTCTTTGATTAACGCTGCCTTCACATTTCAAGGTACT
271 P G I I S K D K N E G R F L G W V S S L I N A A F T F Q G T

G>A G>A G>A C>A A>T -T G>A T>G -C
901 GAACTAGTTGGTATCACTGCTGGTGGAAGCTGCAAACCCCAGAAAATCCGTTCCAAGAGCCATCAAAAAAGTTGTTTTCCGTATCTTAACC
301 E L V G I T A G E A A N P R K S V P R A I K K V V F R I L T

-T -C C>A -T
991 TTCTACATTGGCTCTCTATTATTCATTGGACTTTTAGTTCCATACAATGACCCTAAACTAACACAATCTACTTCCTACGTTTCTACTTCT
331 F Y I G S L L F I G L L V P Y N D P K L T Q S T S Y V S T S

-A A>T -T(2) C>G C>A
1081 CCCTTTATTATTGCTATTGAGAACTCTGGTACAAAGGTTTTGCCACATATCTTCAACGCTGTTATCTTAACAACCATTATTTCTGCCGCA
361 P F I I A I E N S G T K V L P H I F N A V I L T T I I S A A

C>G C>T(2) C>A C>G(2) T>A
1171 AATTCAAATATTTACGTTGGTTCCCGTATTTTATTTGGTCTATCAAAGAACAAGTTGGCTCCTAAATTCCTGTCAAGGACCACCAAAGGT
391 N S N I Y V G S R I L F G L S K N K L A P K F L S R T T K G

G>T A>T
1261 GGTGTTCCATACATTGCAGTTTTCGTTACTGCTGCATTTGGCGCTTTGGCTTACATGGAGACATCTACTGGTGGTGACAAAGTTTTCGAA
421 G V P Y I A V F V T A A F G A L A Y M E T S T G G D K V F E

T>A G>C C>A A>T
1351 TGGCTATTAAATATCACTGGTGTTGCAGGCTTTTTTGCATGGTTATTTATCTCAATCTCGCACATCAGATTTATGCAAGCTTTGAAATAC
451 W L L N I T G V A G F F A W L F I S I S H I R F M Q A L K Y

1441 CGTGGCATCTCTCGTGACGAGTTACCATTTAAAGCTAAATTAATGCCCGGCTTGGCTTATTATGCGGCCACATTTATGACGATCATTATC
481 R G I S R D E L P F K A K L M P G L A Y Y A A T F M T I I I

C>A -T -C(2)
1531 ATTATTCAAGGTTTCACGGCTTTTGCACCAAAAATTCAATGGTGTTAGCTTTGCTGCCGCCTATATCTCTGTTTTCCTGTTCTTAGCTGTT
511 I I Q G F T A F A P K F N G V S F A A A Y I S V F L F L A V

1621 TGGATCTTATTTCAATGCATATTCAGATGCAGATTTATTTGGAAGATTGGAGATGTCGACATCGATTCCGATAGAAGAGACATTGAGGCA
541 W I L F Q C I F R C R F I W K I G D V D I D S D R R D I E A

1711 ATTGTATGGGAAGATCATGAACCAAAGACTTTTTGGGACAAATTTTGGAATGTTGTAGCATAG
571 I V W E D H E P K T F W D K F W N V V A *

Mutation spectrum for CanR colonies derived from 4KR cells

```
                                                                           76-461 (-386bp)
1     ATGACAAATTCAAAAGAAGACGCCGACATAGAGGAGAAGCATATGTACAATGAGCCGGTCACAACCCTCTTTCACGACGTTGAAGCTTCA
1      M  T  N  S  K  E  D  A  D  I  E  E  K  H  M  Y  N  E  P  V  T  T  L  F  H  D  V  E  A  S

      A>T
91    CAAACACACCACAGACGTGGGTCAATACCATTAAAAGATGAGAAAAGTAAAGAATTGTATCCATTGCGCTCTTTCCCGACGAGAGTAAAT
31     Q  T  H  H  R  R  G  S  I  P  L  K  D  E  K  S  K  E  L  Y  P  L  R  S  F  P  T  R  V  N

      G>T(2)
181   GGCGAGGATACGTTCTCTATGGAGGATGGCATAGGTGATGAAGATGAAGGAGAAGTACAGAACGCTGAAGTGAAGAGAGAGCTTAAGCAA
61     G  E  D  T  F  S  M  E  D  G  I  G  D  E  D  E  G  E  V  Q  N  A  E  V  K  R  E  L  K  Q

      +T  G>A(2)  G>C
271   AGACATATTGGTATGATTGCCCTTGGTGGTACTATTGGTACAGGTCTTTTCATTGGTTTATCCACACCTCTGACCAACGCCGGCCCAGTG
91     R  H  I  G  M  I  A  L  G  G  T  I  G  T  G  L  F  I  G  L  S  T  P  L  T  N  A  G  P  V

      C>A  G>C  -CACG  C>A
361   GGCGCTCTTATATCATATTTATTTATGGGTTCTTTGGCATATTCTGTCACGCAGTCCTTGGGTGAAATGGCTACATTCATCCCTGTTACA
121    G  A  L  I  S  Y  L  F  M  G  S  L  A  Y  S  V  T  Q  S  L  G  E  M  A  T  F  I  P  V  T

      C>A  G>A  C>A
451   TCCTCTTTCACAGTGTTCTCACAAAGATTCCTTTCTCCAGCATTTGGTGCGGCCAATGGTTACATGTATTGGTTTTCTTGGGCAATCACT
151    S  S  F  T  V  F  S  Q  R  F  L  S  P  A  F  G  A  A  N  G  Y  M  Y  W  F  S  W  A  I  T

      A>G (2)  -T  C>A
541   TTTGCCCTGGAACTTAGTGTAGTTGGCCAAGTCATTCAATTTTGGACGTACAAAGTTCCACTGGCGGCATGGATTAGTATTTTTTGGGTA
181    F  A  L  E  L  S  V  V  G  Q  V  I  Q  F  W  T  Y  K  V  P  L  A  A  W  I  S  I  F  W  V

      C>A  G>T  A>G  G>T  G>T
631   ATTATCACAATAATGAACTTGTTCCCTGTCAAATATTACGGTGGAATTCGAGTTCTGGGTCGCTTCCATCAAAGTTTTAGCCATTATCGGG
211    I  I  T  I  M  N  L  F  P  V  K  Y  Y  G  E  F  E  F  W  V  A  S  I  K  V  L  A  I  I  G

      duplication(762-810bp)  G>T(2)
      -T  -T  -G  G>T  G>A  -A(2)  -GG
721   TTTCTAATATACTGTTTTTGTATGGTTTGTGGTGCTGGGGTTACCGGCCCAGTTGGATTCCGTTATTGGAGAAACCCAGGTGCCTGGGGT
241    F  L  I  Y  C  F  C  M  V  C  G  A  G  V  T  G  P  V  G  F  R  Y  W  R  N  P  G  A  W  G

      -G(3)  -A  -AG  -G  -G  G>C(2)  -G
811   CCAGGTATAATATCTAAGGATAAAAACGAAGGGAGGTTCTTAGGGTTGGGTTTCCTCTTTGATTAACGCTGCCTTCACATTTCAAGGTACT
271    P  G  I  I  S  K  D  K  N  E  G  R  F  L  G  W  V  S  S  L  I  N  A  A  F  T  F  Q  G  T

      G>C  -G  C>A  C>T-C(2)  -TCC  A>T(2)  -GTT(2)  T>G
901   GAACTAGTTGGTATCACTGCTGGTGAAGCTGCAAACCCCAGAAAATCCGTTCCAAGAGCCATCAAAAAAGTTGTTTTCCGTATCTTAACC
301    E  L  V  G  I  T  A  G  E  A  A  N  P  R  K  S  V  P  R  A  I  K  K  V  V  F  R  I  L  T

      C>G(3)
      C>A  -ATT  G>A  -T  C>A(2)  C>A
991   TTCTACATTGGCTCTCTATTATTCATTGGACTTTTAGTTCCATACAATGACCCTAAACTAACACAATCTACTTCCTACGTTTCTACTTCT
331    F  Y  I  G  S  L  L  F  I  G  L  L  V  P  Y  N  D  P  K  L  T  Q  S  T  S  Y  V  S  T  S

      G>T(2)  -C  -T
1081  CCCTTTATTATTGCTATTGAGAACTCTGGTACAAAGGTTTTGCCACATATCTTCAACGCTGTTATCTTAACAACCATTATTTCTGCCGCA
361    P  F  I  I  A  I  E  N  S  G  T  K  V  L  P  H  I  F  N  A  V  I  L  T  T  I  I  S  A  A

      A>T  C>A  -T  T>G  C>G
1171  AATTCAAATATTTACGTTGGTTCCCGTATTTTATTTGGTCTATCAAAGAACAAGTTGGCTCCTAAATTCCTGTCAAGGACCACCAAAGGT
391    N  S  N  I  Y  V  G  S  R  I  L  F  G  L  S  K  N  K  L  A  P  K  F  L  S  R  T  T  K  G

      C>A  G>T(2)
1261  GGTGTTCCATACATTGCAGTTTTCGTTACTGCTGCATTTGGCGCTTTGGCTTACATGGAGACATCTACTGGTGGTGACAAAGTTTTCGAA
421    G  V  P  Y  I  A  V  F  V  T  A  A  F  G  A  L  A  Y  M  E  T  S  T  G  G  D  K  V  F  E

      G>A  C>A  C>G
1351  TGGCTATTAAATATCACTGGTGTTGCAGGCTTTTTTGCATGGTTATTTATCTCAATCTCGCACATCAGATTTATGCAAGCTTTGAAATAC
451    W  L  L  N  I  T  G  V  A  G  F  F  A  W  L  F  I  S  I  S  H  I  R  F  M  Q  A  L  K  Y

      -C
1441  CGTGGCATCTCTCGTGACGAGTTACCATTTAAAGCTAAATTAATGCCCGGCTTGGCTTATTATGCGGCCACATTTATGACGATCATTATC
481    R  G  I  S  R  D  E  L  P  F  K  A  K  L  M  P  G  L  A  Y  Y  A  A  T  F  M  T  I  I  I

      -G
1531  ATTATTCAAGGTTTCACGGCTTTTGCACCAAAATTCAATGGTGTTAGCTTTGCTGCCGCCTATATCTCTGTTTTCCTGTTCTTAGCTGTT
511    I  I  Q  G  F  T  A  F  A  P  K  F  N  G  V  S  F  A  A  A  Y  I  S  V  F  L  F  L  A  V

      duplication(1626-1648bp)
1621  TGGATCTTATTTCAATGCATATTCAGATGCAGATTTATTTGGAAGATTGGAGATGTCGACATCGATTCCGATAGAAGAGACATTGAGGCA
541    W  I  L  F  Q  C  I  F  R  C  R  F  I  W  K  I  G  D  V  D  I  D  S  D  R  R  D  I  E  A

1711  ATTGTATGGGAAGATCATGAACCAAAGACTTTTTGGGACAAATTTTGGAATGTTGTAGCATAG
571    I  V  W  E  D  H  E  P  K  T  F  W  D  K  F  W  N  V  V  A  *
```

Mutation spectrum for CanR colonies derived from *4KQ rad59* cells

1 ATGACAAATTCAAAAGAAGACGCCGACATAGAGGAGAAGCATATGTACAATGAGCCGGTCACAACCCTCTTTCACGACGTTGAAGCTTCA
1 M T N S K E D A D I E E K H M Y N E P V T T L F H D V E A S

149-491(+342 bp) TGGAATAGCAACTATCA............CATTTCAGAATTGT

C>T -T
91 CAAACACACCACAGACGTGGGTCAATACCATTAAAAGATGAGAAAAGTAAAGAATTGTATCCATTGCGCTCTTTCCCGACGAGAGTAAAT
31 Q T H H R R G S I P L K D E K S K E L Y P L R S F P T R V N

G>T -AG -C
181 GGCGAGGATACGTTCTCTATGGAGGATGGCATAGGTGATGAAGATGAAGGAGAAGTACAGAACGCTGAAGTGAAGAGAGAGCTTAAGCAA
61 G E D T F S M E D G I G D E D E G E V Q N A E V K R E L K Q

G>C G>A -G G>C(2) G>A +C TG G>T C>G
271 AGACATATTGGTATGATTGCCCTTGGTGGTACTATTGGTACAGGTCTTTTCATTGGTTTATCCACACCTCTGACCAACGCCGGCCCAGTG
91 R H I G M I A L G G T I G T G L F I G L S T P L T N A G P V

-C T>C G>C(2) T>A C>A G>C
361 GGCGCTCTTATATCATATTTATTTATGGGTTCTTTGGCATATTCTGTCACGCAGTCCTTGGGTGAAATGGCTACATTCATCCCTGTTACA
121 G A L I S Y L F M G S L A Y S V T Q S L G E M A T F I P V T

G>C -G A>G
451 TCCTCTTTCACAGTGTTCTCACAAAGATTCCTTTCTCCAGCATTTGGTGCGGCCAATGGTTACATGTATTGGTTTTCTTGGGCAATCACT
151 S S F T V F S Q R F L S P A F G A A N G Y M Y W F S W A I T

-G C>T
541 TTTGCCCTGGAACTTAGTGTAGTTGGCCAAGTCATTCAATTTTGGACGTACAAAGTTCCACTGGCGGCATGGATTAGTATTTTTTGGGTA
181 F A L E L S V V G Q V I Q F W T Y K V P L A A W I S I F W V

-A G>C G>T -G G>C
631 ATTATCACAATAATGAACTTGTTCCCTGTCAAATATTACGGTGAATTCGAGTTCTGGGTCGCTTCCATCAAAGTTTTAGCCATTATCGGG
211 I I T I M N L F P V K Y Y G E F E F W V A S I K V L A I I G

-C -T G>A -A(3) -G
721 TTTCTAATATACTGTTTTTGTATGGTTTGTGGTGCTGGGGTTACCGGCCCAGTTGGATTCCGTTATTGGGAGAAACCCAGGTGCCTGGGGT
241 F L I Y C F C M V C G A G V T G P V G F R Y W R N P G A W G

-G -C -G(2) G>T(2) -T-T C>T C>A G>A(2)
811 CCAGGTATAATATCTAAGGATAAAAACGAAGGGAGGTTCTTAGGTTGGGTTTCCTCTTTGATTAACGCTGCCTTCACATTTCAAGGTACT
271 P G I I S K D K N E G R F L G W V S S L I N A A F T F Q G T

G>A G>C G>T C>G -C(3) C>A
901 GAACTAGTTGGTATCACTGCTGGTGAAGCTGCAAACCCCAGAAAATCCGTTCCAAGAGCCATCAAAAAAGTTGTTTTCCGTATCTTAACC
301 E L V G I T A G E A A N P R K S V P R A I K K V V F R I L T

T>C G>A -GG +T T>C -T(2)
991 TTCTACATTGGCTCTCTATTATTCATTGGACTTTTAGTTCCATACAATGACCCTAAACTAACACAATCTACTTCCTACGTTTCTACTTCT
331 F Y I G S L L F I G L L V P Y N D P K L T Q S T S Y V S T S

-G -T
1081 CCCTTTATTATTGCTATTGAGAACTCTGGTACAAAGGTTTTGCCACATATCTTCAACGCTGTTATCTTAACAACCATTATTTCTGCCGCA
361 P F I I A I E N S G T K V L P H I F N A V I L T T I I S A A

C>G T>C G>T C>G -G -G-A
1171 AATTCAAATATTTACGTTGGTTCCCGTATTTTATTTGGTCTATCAAAGAACAAGTTGGCTCCTAAATTCCTGTCAAGGACCACCAAAGGT
391 N S N I Y V G S R I L F G L S K N K L A P K F L S R T T K G

1334-1372 (-39bp)

G>T -T C>G -C G>T
1261 GGTGTTCCATACATTGCAGTTTTCGTTACTGCTGCATTTGGCGCTTTGGCTTACATGGAGACATCTACTGGTGGTGACAAAGTTTTCGAA
421 G V P Y I A V F V T A A F G A L A Y M E T S T G G D K V F E

1398-1737 (+339 bp)GAGAAATATGTGAA.........CTATTCCAACAGTTATT

G>C C>G A>T C>T G>C A>T
1351 TGGCTATTAAATATCACTGGTGTTGCAGGCTTTTTTGCATGGTTATTTATCTCAATCTCGCACATCAGATTTATGCAAGCTTTGAAATAC
451 W L L N I T G V A G F F A W L F I S I S H I R F M Q A L K Y

-ATG +TT
1441 CGTGGCATCTCTCGTGACGAGTTACCATTTAAAGCTAAATTAATGCCCGGCTTGGCTTATTATGCGGCCACATTTATGACGATCATTATC
481 R G I S R D E L P F K A K L M P G L A Y Y A A T F M T I I I

C>A
1531 ATTATTCAAGGTTTCACGGCTTTTGCACCAAAATTCAATGGTGTTAGCTTTGCTGCCGCCTATATCTCTGTTTTCCTGTTCTTAGCTGTT
511 I I Q G F T A F A P K F N G V S F A A A Y I S V F L F L A V

-ATG(2) +TT(2)
1621 TGGATCTTATTTCAATGCATATTCAGATGCAGATTTATTTGGAAGATTGGAGATGTCGACATCGATTCCGATAGAAGAGACATTGAGGCA
541 W I L F Q C I F R C R F I W K I G D V D I D S D R R D I E A

1711 ATTGTATGGGAAGATCATGAACCAAAGACTTTTTGGGACAAATTTTGGAATGTTGTAGCATAG
571 I V W E D H E P K T F W D K F W N V V A *

Figure S7 continued

Mutation spectrum for CanR colonies derived from *4KQ rtt105-EL2A* cells

15-1178 (-1164bp)

1 ATGACAAATTCAAAAGAAGACGCCGACATAGAGGAGAAGCATATGTACAATGAGCCGGTCACAACCCTCTTTCACGACGTTGAAGCTTCA
1 M T N S K E D A D I E E K H M Y N E P V T T L F H D V E A S

C>G

91 CAAACACACCACAGACGTGGGTCAATACCATTAAAAGATGAGAAAAGTAAAGAATTGTATCCATTGCGCTCTTTCCCGACGAGAGTAAAT
31 Q T H H R R G S I P L K D E K S K E L Y P L R S F P T R V N

-A A>G

181 GGCGAGGATACGTTCTCTATGGAGGATGGCATAGGTGATGAAGATGAAGGAGAAGTACAGAACGCTGAAGTGAAGAGAGAGCTTAAGCAA
61 G E D T F S M E D G I G D E D E G E V Q N A E V K R E L K Q

297-421 (-125bp)
G>T C>G -C

271 AGACATATTGGTATGATTGCCCTTGGTGGTACTATTGGTACAGGTCTTTTCATTGGTTTATCCACACCTCTGACCAACGCCGGCCCAGTG
91 R H I G M I A L G G T I G T G L F I G L S T P L T N A G P V

-T C>A

361 GGCGCTCTTATATCATATTTATTTATGGGTTCTTTGGCATATTCTGTCACGCAGTCCTTGGGTGAAATGGCTACATTCATCCCTGTTACA
121 G A L I S Y L F M G S L A Y S V T Q S L G E M A T F I P V T

533-628 (-96bp)

451 TCCTCTTTCACAGTGTTCTCACAAAGATTCCTTTCTCCAGCATTTGGTGCGGCCAATGGTTACATGTATTGGTTTTCTTGGGCAATCACT
151 S S F T V F S Q R F L S P A F G A A N G Y M Y W F S W A I T

-T
+AA
-AT
G>A -T(2)

541 TTTGCCCTGGAACTTAGTGTAGTTGGCCAAGTCATTCAATTTTGGACGTACAAAGTTCCACTGGCGGCATGGATTAGTATTTTTTGGGTA
181 F A L E L S V V G Q V I Q F W T Y K V P L A A W I S I F W V

-G

631 ATTATCACAATAATGAACTTGTTCCCTGTCAAATATTACGGTGAATTCGAGTTCTGGGTCGCTTCCATCAAAGTTTTAGCCATTATCGGG
211 I I T I M N L F P V K Y Y G E F E W V A S I K V L A I I G

762-810 (-49bp)
G>C
G>T(2) -A G>A G>A -G G>T

721 TTTCTAATATACTGTTTTTGTATGGTTTGTGGTGCTGGGGTTACCGGCCAGTTGGATTCCGTTATTGGAGAAACCCAGGTGCCTGGGGT
241 F L I Y C F C M V C G A G V T G P V G F R Y W R N P G A W G

G>C
G>C -G(3) -C-G G>A G>T A>C -C G>T

811 CCAGGTATAATATCTAAGGATAAAAACGAAGGGAGGTTCTTAGGGTTGGGTTTCCTCTTTGATTAACGCTGCCTTCACATTTCAAGGTACT
271 P G I I S K D K N E G R F L G W V S S L I N A A F T F Q G T

G>C T>G
G>T T>C -G C>A A>T -TC -A -C

901 GAACTAGTTGGTATCACTGCTGGTGAAGCTGCAAACCCCAGAAAATCCGTTCCAAGAGCCATCAAAAAAGTTGTTTTCCGTATCTTAACC
301 E L V G I T A G E A A N P R K S V P R A I K K V V F R I L T

991 TTCTACATTGGCTCTCTATTATTCATTGGACTTTTAGTTCCATACAATGACCCTAAACTAACACAATCTACTTCCTACGTTTCTACTTCT
331 F Y I G S L L F I G L L V P Y N D P K L T Q S T S Y V S T S

G>C

1081 CCCTTTATTATTGCTATTGAGAACTCTGGTACAAAGGTTTTGCCACATATCTTCAACGCTGTTATCTTAACAACCATTATTTCTGCCGCA
361 P F I I A I E N S G T K V L P H I F N A V I L T T I I S A A

1195-1233 (-38bp)
C>G C>A +T C>T -G

1171 AATTCAAATATTTACGTTGGTTCCCGTATTTTATTTGGTCTATCAAAGAACAAGTTGGCTCCTAAATTCCTGTCAAGGACCACCAAAGGT
391 N S N I Y V G S R I L F G L S K N K L A P K F L S R T T K G

-GTG
+TT
G>T C>G Duplication (1279-1296) A>T -C

1261 GGTGTTCCATACATTGCAGTTTTCGTTACTGCTGCATTTGGCGCTTTGGCTTACATGGAGACATCTACTGGTGGTGACAAAGTTTTCGAA
421 G V P Y I A V F V T A A F G A L A Y M E T S T G G D K V F E

T>A C>G C>G A>T

1351 TGGCTATTAAATATCACTGGTGTTGCAGGCTTTTTTGCATGGTTATTTATCTCAATCTCGCACATCAGATTTATGCAAGCTTTGAAATAC
451 W L L N I T G V A G F F A W L F I S I S H I R F M Q A L K Y

1447-1641 (-195bp)
-ACG
T>A T>A -A -C(1) -A(1) +CC

1441 CGTGGCATCTCTCGTGACGAGTTACCATTTAAAGCTAAATTAATGCCCGGCTTGGCTTATTATGCGGCCACATTTATGACGATCATTATC
481 R G I S R D E L P F K A K L M P G L A Y Y A A T F M T I I I

A>G

1531 ATTATTCAAGGTTTCACGGCTTTTGCACCAAAATTCAATGGTGTTAGCTTTGCTGCCGCCTATATCTCTGTTTTCCTGTTCTTAGCTGTT
511 I I Q G F T A F A P K F N G V S F A A Y I S V F L F L A V

+TT
C>T ATG

1621 TGGATCTTATTTCAATGCATATTCAGATGCAGATTTATTTGGAAGATTGGAGATGTCGACATCGATTCCGATAGAAGAGACATTGAGGCA
541 W I L F Q C I F R C R F I W K I G D V D I D S D R R D I E A

1711 ATTGTATGGGAAGATCATGAACCAAAGACTTTTTGGGACAAATTTTGGAATGTTGTAGCATAG
571 I V W E D H E P K T F W D K F W N V V A *

Supplementary Figure S7. Mutation spectrum of the *CAN1* gene for indicated yeast strains. CanR isolates were randomly picked and sequenced for the *CAN1* gene. Base substitution, deletion, insertion and duplication events are marked. Arrows mark the positions where large deletion or insertion events occurred. The exact positions and lengths of deleted or duplicated sequences are indicated. The deleted sequences are marked in red, while the duplicated sequences are marked in blue and underlined. The numbers in brackets represent the frequency of observation of the same event.

Figure S8

<u>insertion (1398-1737, +339 bps) of YPRdelta18 sequence:</u>

TTTGCATG<u>***GTTATT***</u>GAGAAATATGTGAATGTTGAGATAATTGTTGGGATTCCATTGTTGATAAAGGCTATAATATTAGGTATACAG
AATATACTAGAAGTTCTCCTCAAGGGTTTAGGAATCCATAAAAGGGAATCTGCAATTCTACACAATTCTATAAATATTATTATCAT
CATTTTATATGTTTATATTCATTGATCCTATTACATTATCAATCCTTGCGTTTCAGCTTCCACTAATTTAGATGACTATTTCTCATCA
TTTGCGTCATCTTCTAACACCGTATATGATAATATACTAGTAATGTAAATACTAGTTAGTAGATGATAGTTGATTTCTATTCCAACA
<u>***GTTATT***</u>TATCTCAA

<u>insertion (149-491, +342 bps) of YCLWdelta15 sequence:</u>

GAAAAGTA<u>***AAGAATTGT***</u>TGGAATAGAAATCAACTATCATCTACTAACTAGTATTTACATTACTAGTATATTATCATATACGGTGTT
AGAAGATGACGCAAATGATGAGAAATAGTCATCTAAATTAGTGGAAGCTGAAACGCAAGGATTGATAATGTAATAGGATCAATG
AATATAAACATATAAAACGGAATGAGGAATAATCGTAATATTAGTATGTAGAAATATAGATTCCATTTTGAGGATTCCTATATCCT
CGAGGAGAACTTCTAGTATATTCTGTATACCTAATATTATAGCCTTTATCAACAATGGAATCCCAACAATTATCTCAACATTCACC
CATTTCTC<u>***AAGAATTGT***</u>ATCCATTG

Supplementary Figure S8. The sequence and position of Ty1 retrotransposon inserted in the *CAN1* gene in the *4KQ rad59*△ mutant cells. Micro-homologies are underlined. Inserted sequences are marked in red.

Figure S9

Strain	Mutation type	Sequence	Position	Size (bp)	Micro-homology (bp)
4KQ (4/85)	Deletion (4/85)	TGGTTTTCTTGGGCAATCACTTT……….G<br>TATTTTTTGGGTAATTATC	533-628	96	5
		TTTATGGGTTCTTTGGCATATT……..GTAT<br>TGGTTTTCTTGGGCAATC	395-529	135	5
		TTATGGGTTCTTTGGCATATTCTG……….C<br>ATCCTCTTTCACAGTTT	396-458	63	5
		TGGTTTTCTTGGGCAATCACTTT……….G<br>TATTTTTTGGGTAATTATC	533-628	96	5
4KR (3/85)	Deletion (1/85)	TCTTTCACCCTCTTTCACGAC……..ACAT<br>CCTCTTTCACAGTGTTCT	76-461	386	10
	Duplication (2/85)	TTGTGGTGCTGGGGTTGGAG……..AGGT<br>GCCTGGGGTCCAGGTAT	762-810	49	7
		GCTGTTTGGATCTTAT………..TTCAGATG<br>CAGATTTATTTGGAAGA	1626-1648	23	3
4KQ *rad59Δ* (3/96)	Deletion (1/96)	AGACATCTACTGGTGGTGACAA…AATAT<br>CACTGGTGTTGCAGGC	1334-1372	39	7
	Insertion (2/96)	TTTGCATGGTTATTGAGAAA……………..C<br>AACAGTTATTTATCTCAA	1398-1737	339	6
		GAAAAGTAAAGAATTGTTGGAA……TCT<br>CAAGAATTGTATCCATTG	149-491	342	9
4KQ/*rtt105-EL2A* (7/84)	Deletion (6/84)	TGGTTTTCTTGGGCAATCACTT……ATTA<br>GTATTTTTTGGGTAATTATC	533-628	96	5
		ACGTTGGTTCCCGTATTTTATTTGCAA…C<br>AAGTTGGCTCCTAAATTCC	1195-1232	38	3
		ATGATTGCCCTTGGTGGTACTAT……….A<br>CGCAGTCCTTGGGTGAAA	297-421	125	6
		TTGTGGTGCTGGGGTTACCGGCA………C<br>CGTGCCTGGGGTCCAGG	762-810	49	7
		ATGACAAATTCAAAAGAAGACGC……..C<br>TGCCGCAAATTCAAATATTT	15-1178	1164	10
		GCTTTGAAATACCGTGGCATCTCTC……<br>……TTTCAATGCATATTCAGA	1447-1641	195	3
	Duplication (1/84)	ACATTGCATGCAGTTTTCGTTACTGCTGC<br>ATTTGGCGC	1279-1296	18	4

Supplementary Figure S9. Table listing the duplication or deletion events mediated by micro-homologies in indicated strains. The deleted or duplicated sequences are marked in red, and the flanking short homologies are marked in gray shadow. The sizes for the duplication, deletion or micro-homologies are indicated.

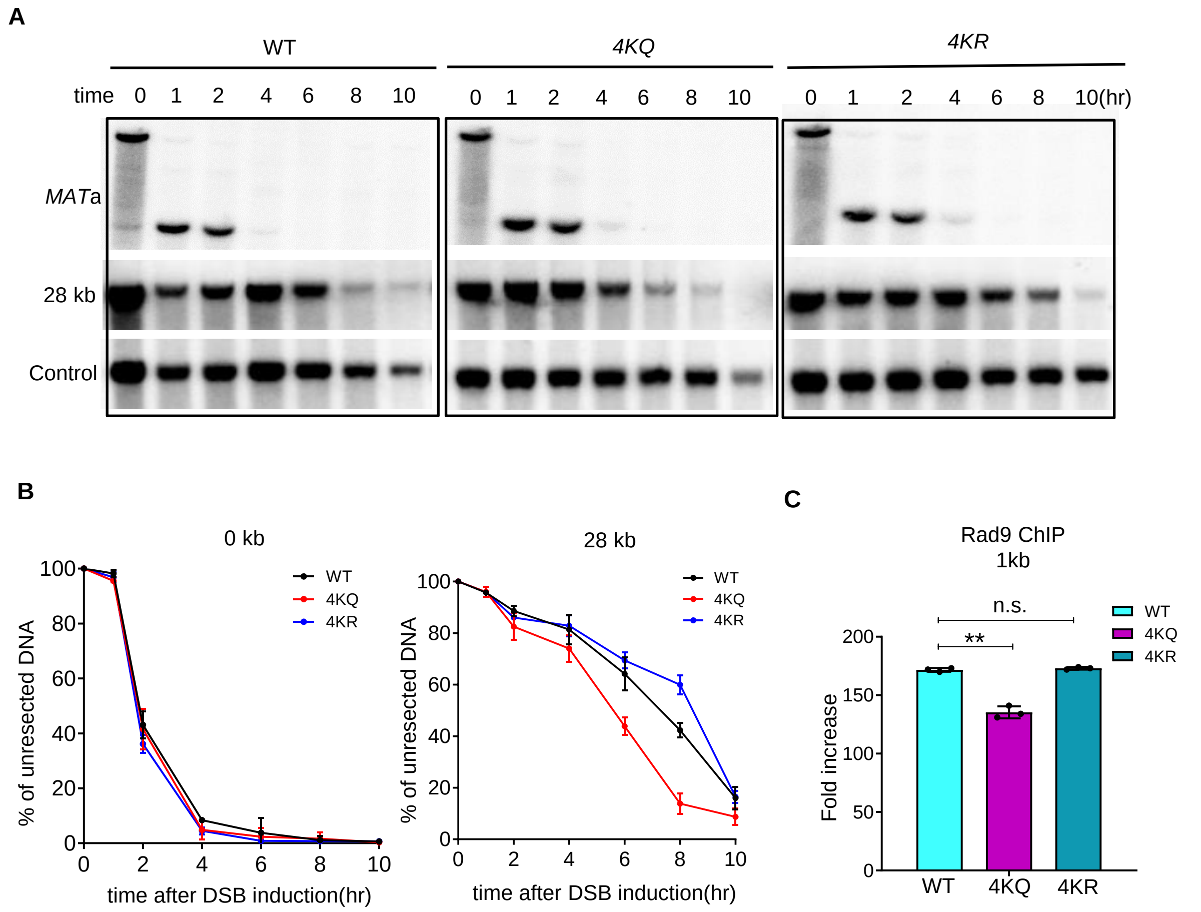

Supplementary Figure S10. The impact of dynamic RPA acetylation on DNA end resection and Rad9 recruitment. **A.** Southern blot analysis of DSB end resection for indicated cells. Samples were collected at indicated time points after DSB induction. The blot was hybridized with the *MAT*a, 28 kb, or the control *TRA1* probe. **B.** Quantification of the results in (A). **C.** ChIP-qPCR showing the enrichment of Rad9-3xFLAG at 1kb away from the DSB in indicated strains. Samples were collected at 4hr after DSB induction. Error bars represent standard deviations from three independent experiments. Statistical analysis was calculated with the Student *t*-test. ** $p < 0.01$, n.s. no significance.

Figure S11

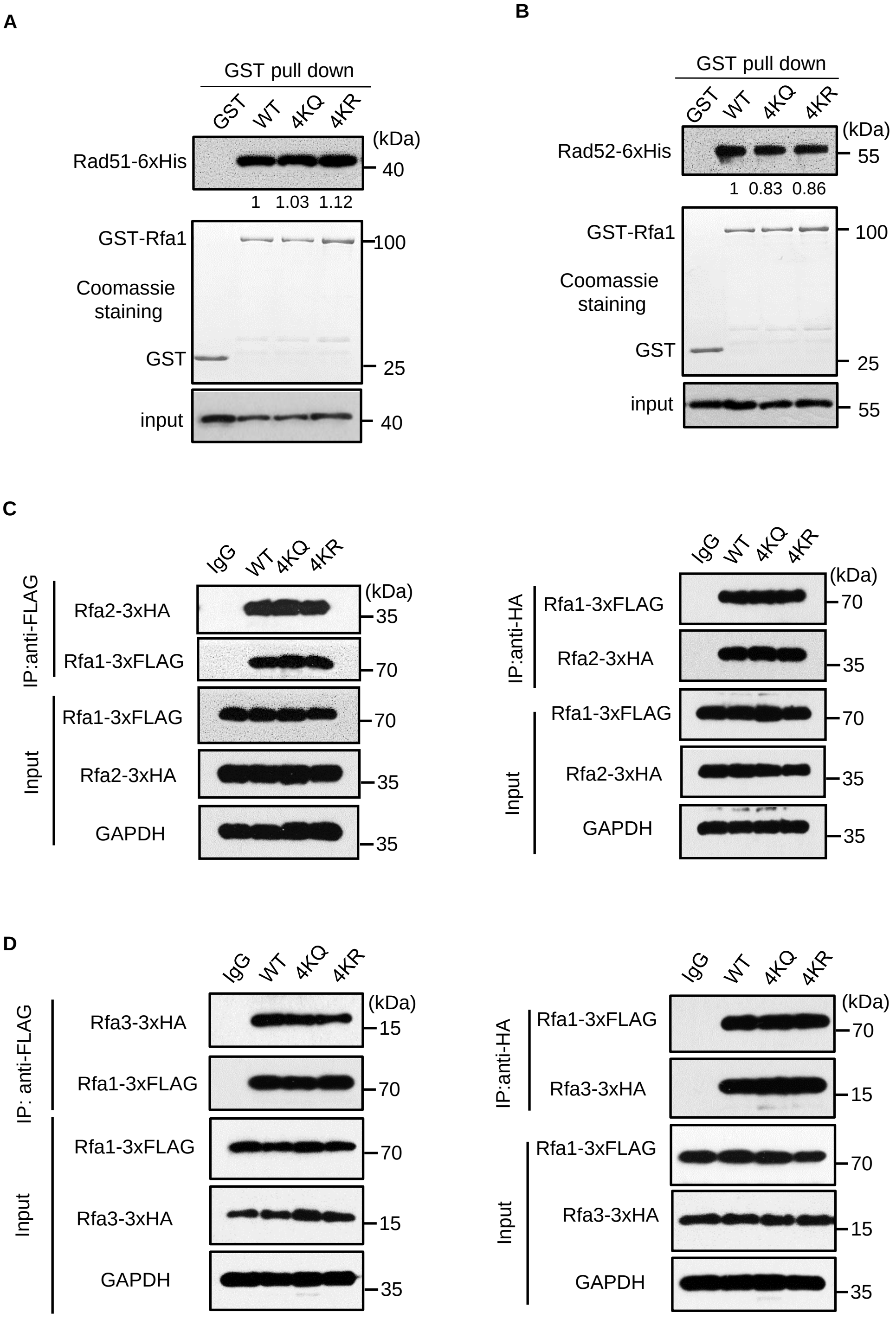

Supplementary Figure S11. The 4KQ or 4KR mutation does not affect the RPA complex formation or its interaction with Rad51 or Rad52. A-B. GST pull-down assay showing interactions between the GST-tagged WT or mutant Rfa1 and 6xHis-Rad51 or 6xHis-Rad52 in vitro. GST was used as a control. The amount of Rfa1 protein used is indicated by Coomassie blue staining. The relative amount of Rad51 or Rad52 associated with RPA is indicated by the numbers. **C.** Co-immunoprecipitation and Western blot analysis showing the interaction between Rfa1-3xFLAG and Rfa2-3xHA in indicated cells. **D.** Co-immunoprecipitation showing the interaction between Rfa1-3xFLAG and Rfa3-3xHA in indicated cells.

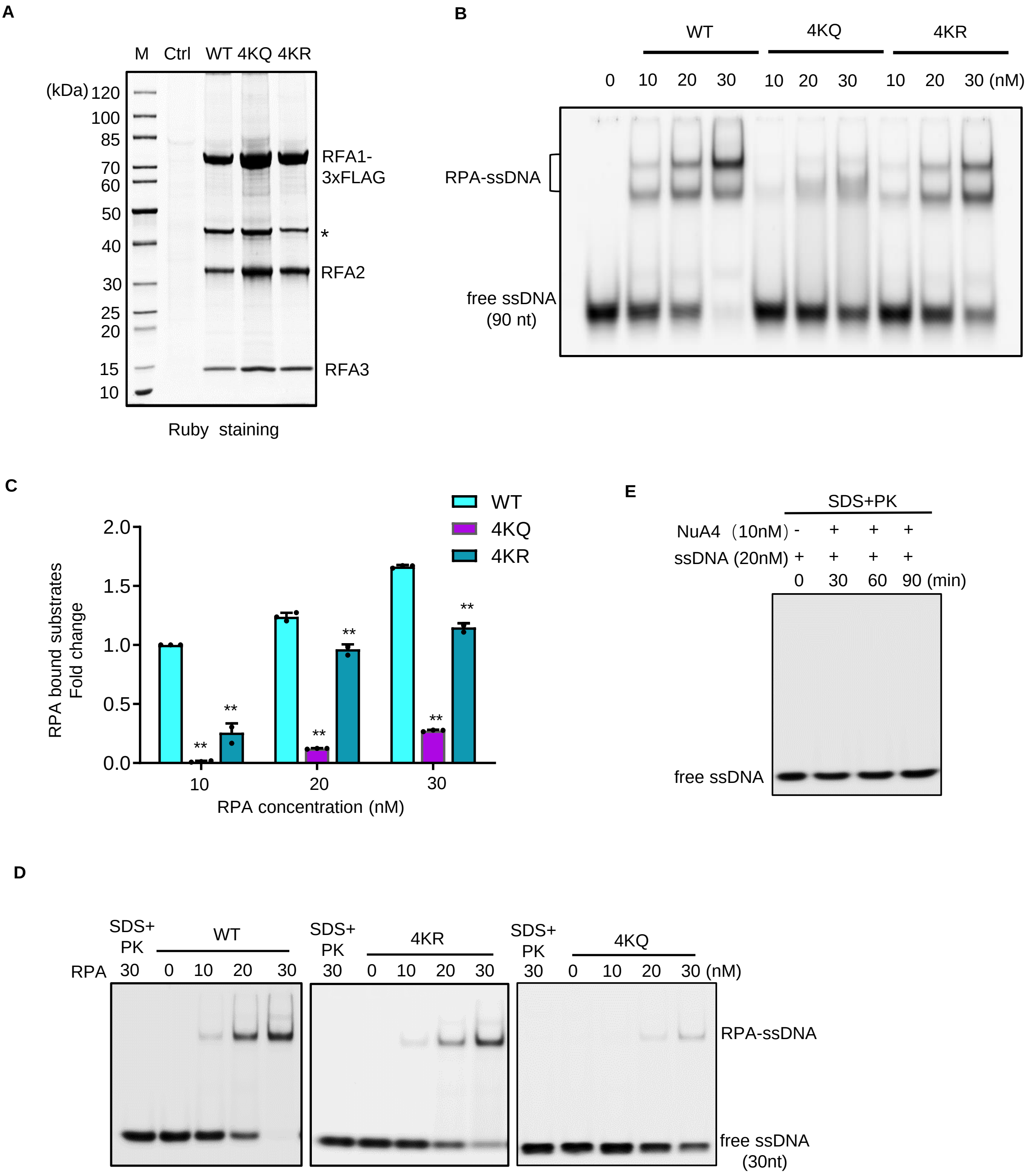

Supplementary Figure S12. Proper acetylation and deacetylation of RPA are required for efficient RPA binding to ssDNA. A. SDS-PAGE and Coomassie blue staining of the RPA complex purified from yeast cells. **B.** An EMSA assay showing the binding of the purified WT, 4KQ or 4KR RPA to ssDNA (90 nt). Protein concentrations are indicated. **C.** Quantification of the relative ssDNA binding ability for indicated proteins presented in B. Error bars represent standard deviations from three independent experiments. Statistical analysis was calculated with the Student *t*-test. ** $p < 0.01$. **D.** EMSA assays showing the binding of the purified WT, 4KQ or 4KR RPA to ssDNA in the presence or absence of SDS/protease K treatment (1%SDS, 1mg/ml protease K, 37℃, 30 mins). **E.** An EMSA assay showing that incubation of the purified NuA4 complex with ssDNA does not cause DNA degradation.

Figure S13

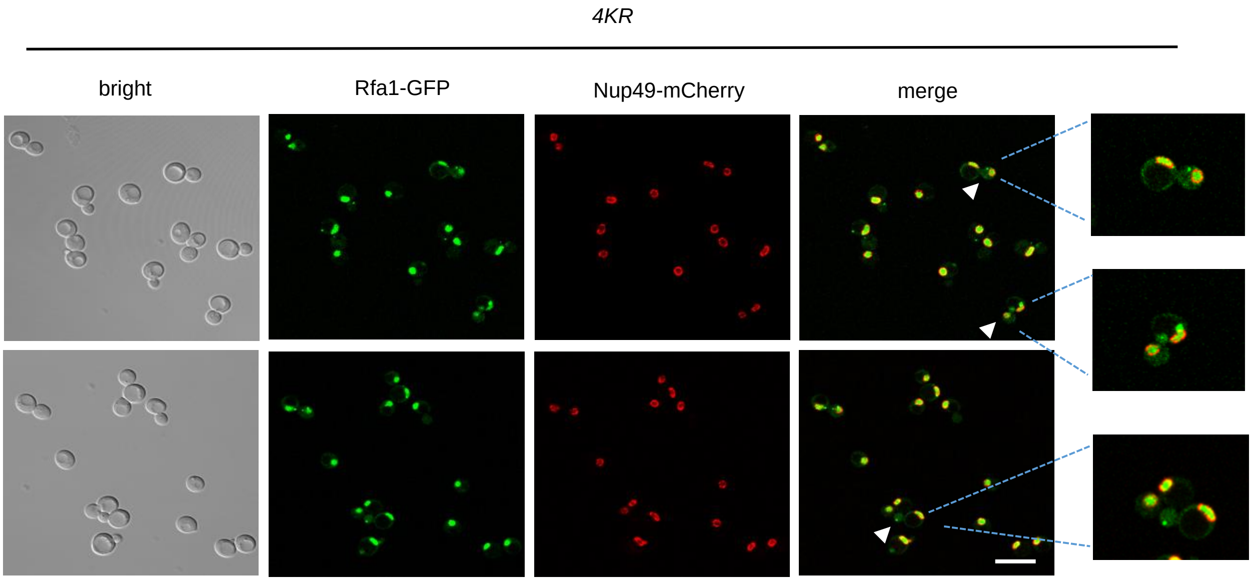

Supplementary Figure S13. Microscopy analysis of RPA nuclear localization in *4KR* mutant cells. Rfa1 was tagged with a YFP at the C-terminus, while the nuclear envelope protein Nup49 was fused with a mCherry at the C-terminus. Arrows indicate the *4KR* cells with changed Rfa1 subcellular localization.

Figure S14

A

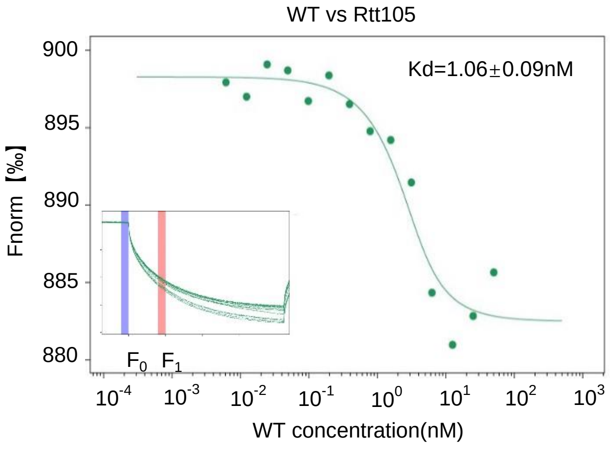

B

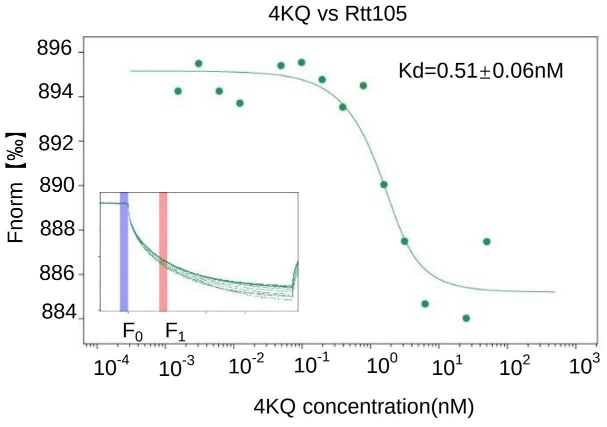

C

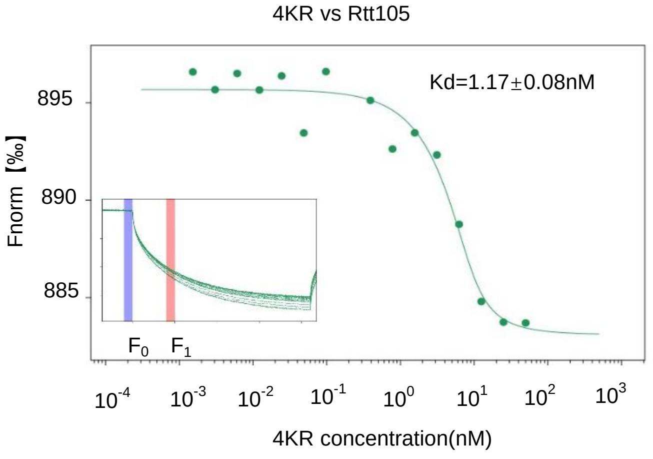

Supplementary Figure S14. The binding affinity of the WT or mutant RPA protein to Rtt105. The in vitro binding affinity between 6xHis-Rtt105 and the WT, 4KQ or 4KR mutant RPA was tested by the MST assay. Inset, thermophoretic movement of fluorescently labeled proteins. Fnorm = F1/F0 (Fnorm: normalized fluorescence; F1: fluorescence after thermodiffusion; F0: initial fluorescence or fluorescence after T-jump). Kd, dissociation constant.

Figure S15

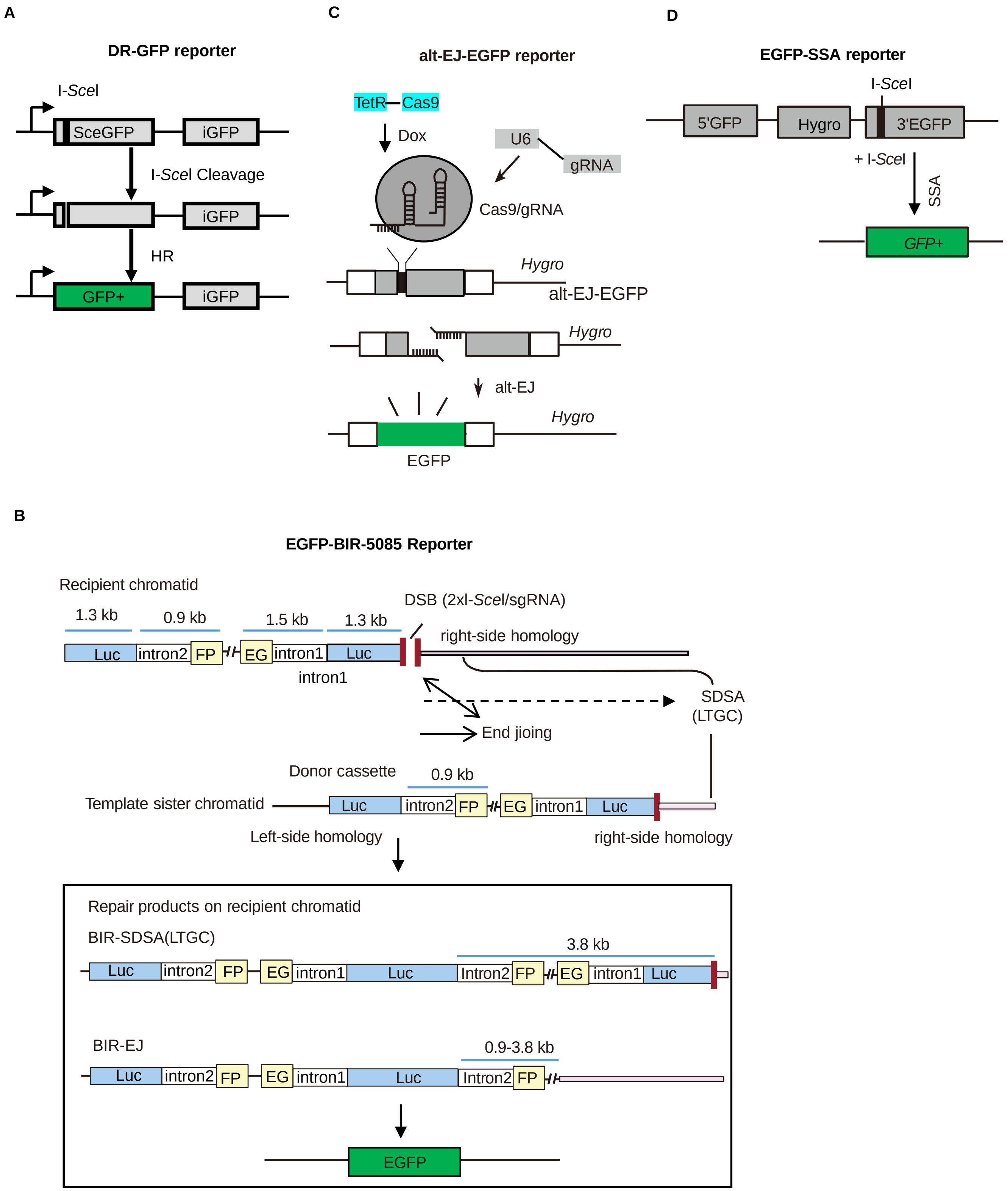

Supplementary Figure S15. Graphs showing different reporter assays for DSB repair used in this study. (A) Schematic representation of the EGFP-based HR reporter assay. **(B)** Schematic representation of the EGFP-based BIR reporter assay. **(C)** Schematic representation of the EGFP-based alt-EJ reporter assay. **(D)** Schematic representation of the EGFP-based SSA reporter assay.

Figure S16

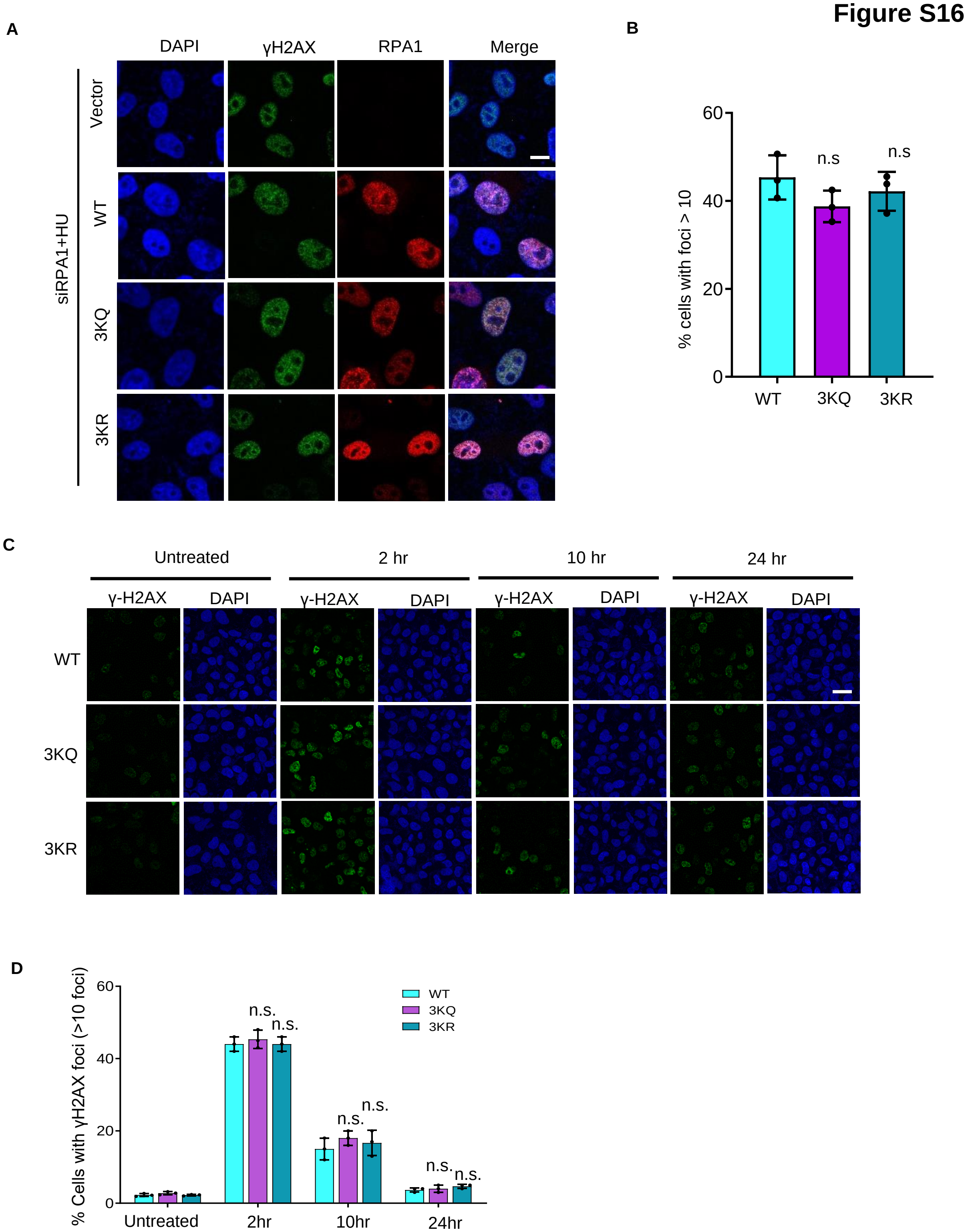

Supplementary Figure S16. Proper acetylation and deacetylation of RPA are not required for the response to the HU-induced replication stresses. A. Immunostaining of the HU-induced γH2AX and RPA foci in indicated cell lines. Scale bar: 10 μm. **B.** Quantification of (A) is the average of three independent experiments. Over 100 cells were counted for each experiment. **C**. Immunostaining showing the formation and removal of γH2AX foci following HU treatment in indicated cells. Cells were treated with 5 mM HU for 4hrs before releasing to media without HU. Samples were collected at indicated time points following HU treatment. Untreated cells were used as controls. **D.** Quantification of γH2AX foci in indicated cells at indicated time points. Error bars represent standard deviations from three independent experiments. Statistical analysis was calculated with the Student *t*-test. n.s., no significance.

Figure S17

A

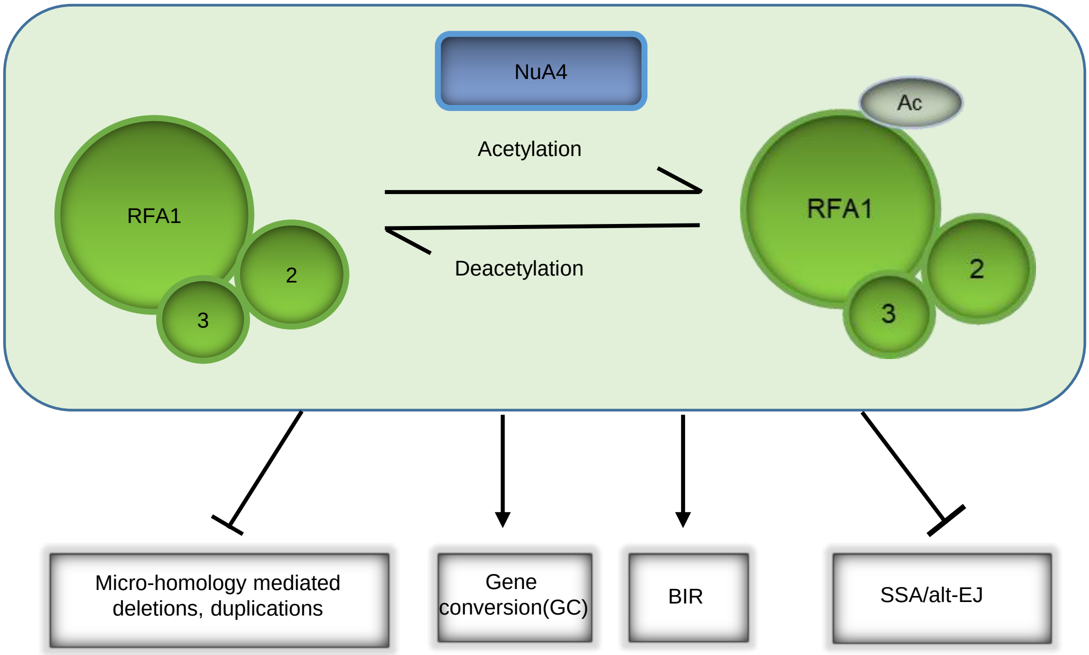

B

		GC	BIR	SSA	alt-EJ
Yeast	4KQ	+	+	+	+
	4KR	+	-	+	+
Human	3KQ	+	+	+	+
	3KR	+	-	-	-

Supplementary Figure S17. A. A model showing the role of proper RPA acetylation and deacetylation in promoting high-fidelity DNA replication and repair. **B.** The impact of RPA acetylation/deacetylation on different DSB repair pathways. "+" , affected; "-", unaffected.

Supplementary Table 1. Yeast strains

Strain name	Parental strain	Genotype	Source
tGI354		*MATa-inc arg5,6::MATa-HPHMX,ade3::GAL::HO hmr::ADE1 hml::ADE1 ura3-52*	Ira et al (2003)
JKM139		*MATa ho hml::ADE1 hmr::ADE1 ade1-100 leu2-3,112 trp1::hisG lys5 ura3-52 ade3::GAL::HO*	Lee et al(1998)
GC1	BY4742	*MATα his3Δ200 ura3Δ0 met15Δ0 TRP1 Δ63/YAC (MFA1pr-HIS3 URA3 MET15 TRP1)*	Wahba et al (2011)
NA29	MK203	*MATa-inc ura3-HOcs lys2::ura3-HOcs-inc ade3::GALHO ade2-1 leu2-3,112 his3-11,15 trp1-1 can1-100*	Aylon et al(2002)
yWH378		*ho hml ::ADE1 mata ::hisG hmr ::ADE1 his4::NatMX leu2-(XhoI- to Asp718) leu2::MATa ade3::GAL::HO ade1 lys5 ura3-52 trp1 rad51::URA3*	Zhu et al (2008)
AM1003		*MATa-LEU2-tel/MATa-inc ade1 met13 ura3 leu2-3,112/leu2 thr4 lys5 hml::ADE1/hml::ADE3 hmr::HYG ade3::GAL-HO FS2::NAT/FS2*	Deem et al (2008)
yXL105	JKM139	*rfa1-4KQ-TRP1*	This study
yXL106	JKM139	*rfa1-4KR-TRP1*	This study
yXL111	JKM139	*ku70::NatMX rfa1-4KR-TRP1*	This study
yXL112	JKM139	*ku70::NatMX rfa1-4KQ-TRP1*	This study
yXL071	JKM139	*rfa1-3KR-3xFlag-NatMX*	This study
yXL092	JKM139	*rfa1-4KQ-3xFlag-NatMX*	This study
yXL093	JKM139	*rfa1-4KR-3xFlag-NatMX*	This study
yXL099	JKM139	*Rad53-3xFlag-KanMX rfa1-4KQ-TRP1*	This study
yXL100	JKM139	*Rad53-3xFlag-KanMX rfa1-4KR-TRP1*	This study
yXL162	JKM139	*Rad9-3xFlag-NatMX rfa1-4KQ-TRP1*	This study
yXL163	JKM139	*Rad9-3xFlag-NatMX rfa1-4KR-TRP1*	This study
yXL107	JKM139	*rtt105-EL2A-KanMX rfa1-4KQ-TRP1*	This study
yXL103	JKM139	*Rad51-3xFlag-KanMX rfa1-4KR-TRP1*	This study
yXL104	JKM139	*Rad51-3xFlag-KanMX rfa1-4KQ-TRP1*	This study
yXL101	JKM139	*Rad52-13xmyc-HPHMX rfa1-4KR-TRP1*	This study
yXL102	JKM139	*Rad52-13xmyc-HPHMX rfa1-4KQ-TRP1*	This study
yXL109	JKM139	*rfa1-4KR-YFP-HPHMX Nup49-mCherry-TRP1*	This study
yXL110	JKM139	*rfa1-4KQ-YFP-HPHMX Nup49-mCherry-TRP1*	This study
yXL084	JKM139	*RFA3-3xHA-TRP1 RFA1-3xFlag-HPHMX*	This study
yXL085	JKM139	*RFA3-3xHA-TRP1 rfa1-4KR-3xFlag-KanMX*	This study
yXL086	JKM139	*RFA3-3xHA-TRP1 rfa1-4KQ-3xFlag-KanMX*	This study
yXL117	JKM139	*RFA2-3xHA-TRP1 rfa1-4KQ-3xFlag-KanMX*	This study
yXL118	JKM139	*RFA2-3xHA-TRP1 RFA1-3xFlag-HPHMX*	This study
yXL119	JKM139	*RFA2-3xHA-TRP1 rfa1-4KR-3xFlag-KanMX*	This study
yXL120	JKM139	*rfa1-4KR-3xFlag-KanMX*	This study
yXL121	JKM139	*rfa1-4KQ-3xFlag-KanMX*	This study
yXL150	JKM139	*rtt105-EL2A-KanMX*	This study
yXL152	JKM139	*rfa1-4KR-3xFlag-KanMX RTT105-3xHA-TRP1*	This study
yXL153	JKM139	*rfa1-4KQ-3xFlag-KanMX RTT105-3xHA-TRP1*	This study
yXL155	JKM139	*RFA1-3xFlag-NatMX RTT105-3xHA-TRP1*	This study
yXL132	JKM139	*Rad59::KanMX rfa1-4KQ-TRP1*	This study
yXL096	JKM139	*Kap95-3xHA-TRP1 rfa1-4KR-3xFlag-KanMX*	This study
yXL097	JKM139	*Kap95-3xHA-TRP1 RFA1-3xFlag-HPHMX*	This study
yXL098	JKM139	*Kap95-3xHA-TRP1 rfa1-4KQ-3xFlag-KanMX*	This study
yXL081	JKM139	*RFA1-3xFlag-NatMX esa1-L254P-TRP1*	This study
YXL146	GC1	*rfa1-4KQ-KanMX*	This study
yXL145	GC1	*rfa1-4KR-KanMX*	This study
yXL094	tGI354	*rfa1-4KR-TRP1*	This study
yXL095	tGI354	*rfa1-4KQ-TRP1*	This study
yXL151	tGI354	*rtt105-EL2A-KanMX*	This study
yXL149	tGI354	*rfa1-4KQ-TRP1 rtt105-EL2A-KanMX*	This study
yXL174	NA29	*rfa1-4KQ-TRP1*	This study
yXL175	NA29	*rfa1-4KR-TRP1*	This study
yXL113	AM1003	*rfa1-4KQ-KanMX*	This study
yXL114	AM1003	*rfa1-4KR-KanMX*	This study
yXL141	yWH378	*RFA1-4KR-TRP1*	This study
yXL142	yWH378	*RFA1-4KQ-TRP1*	This study
